# Solvent-induced electrochemistry at an electrically asymmetric carbon Janus particle

**DOI:** 10.1038/s41467-021-23038-7

**Published:** 2021-06-07

**Authors:** Albert Tianxiang Liu, Yuichiro Kunai, Anton L. Cottrill, Amir Kaplan, Ge Zhang, Hyunah Kim, Rafid S. Mollah, Yannick L. Eatmon, Michael S. Strano

**Affiliations:** 1 Department of Chemical Engineering, Massachusetts Institute of Technology, Cambridge, MA USA; 2 Present Address: Department of Molecular and Cellular Physiology, Stanford University School of Medicine, Palo Alto, CA 94305 USA

**Keywords:** Electrocatalysis, Energy, Electrocatalysis, Nanoscale materials

## Abstract

Chemical doping through heteroatom substitution is often used to control the Fermi level of semiconductor materials. Doping also occurs when surface adsorbed molecules modify the Fermi level of low dimensional materials such as carbon nanotubes. A gradient in dopant concentration, and hence the chemical potential, across such a material generates usable electrical current. This opens up the possibility of creating asymmetric catalytic particles capable of generating voltage from a surrounding solvent that imposes such a gradient, enabling electrochemical transformations. In this work, we report that symmetry-broken carbon particles comprised of high surface area single-walled carbon nanotube networks can effectively convert exothermic solvent adsorption into usable electrical potential, turning over electrochemical redox processes in situ with no external power supply. The results from ferrocene oxidation and the selective electro-oxidation of alcohols underscore the potential of solvent powered electrocatalytic particles to extend electrochemical transformation to various environments.

## Abstract (web summary)

Chemical doping of low dimensional materials by surface adsorbed molecules has proven to be a source of electrical energy. Here, the authors find that asymmetric particles consisting of carbon nanotubes can drive electrochemical reactions by electrical potential generated from solvent adsorption.

## Introduction

**Fig. 1:**
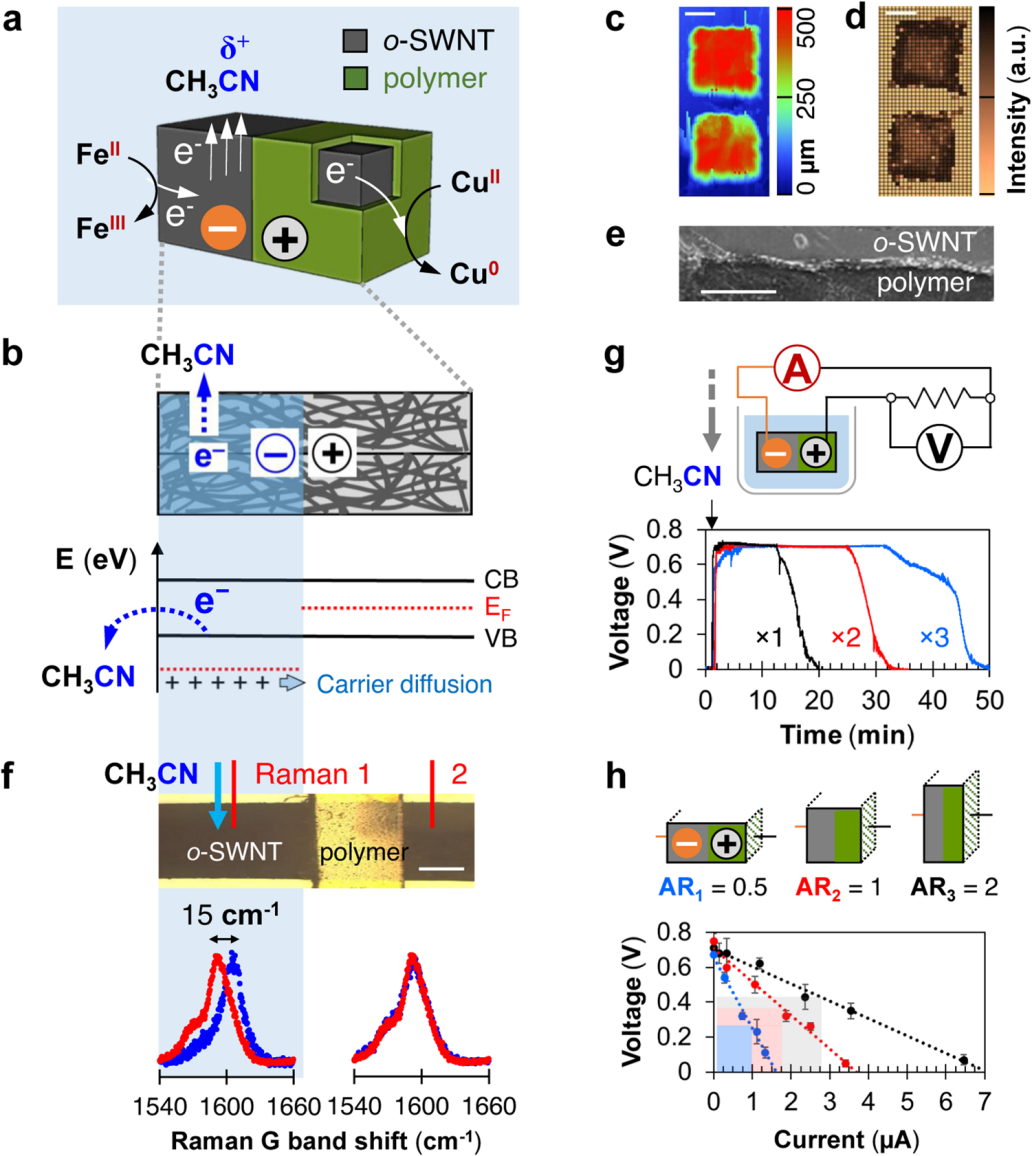
A particle capable of inducing electric potentials from within a solvent. **a** Schematic illustration of Janus microparticles generating electricity to power electrochemical redox reactions (e.g., Fe^2+^ → Fe^3+^ or Cu^2+^ → Cu^0^) in situ, in lieu of a potentiostat as a voltage source. **b** Schematic illustration of the electricity generation mechanism of the Janus microparticle. Asymmetric chemical doping is realized via spatially asymmetric polymer coating. The partial electron transfer induced by CH_3_CN molecules adsorbed on the o-SWNT surface withdraws electron density from the adsorption site, thereby lowering the corresponding Fermi energy (*E*_F_), drawing electron flow following the prescribed *E*_F_ gradient^15^. **c** Surface profile of two 500 µm × 250 µm × 250 µm o-SWNT/PTFE Janus particles (color bar range, 0–500 µm; scale bar, 100 µm). **d** Raman spectroscopic map showing the intensity of the G band of the o-SWNT side of two 500 µm × 250 µm × 250 µm o-SWNT/PTFE Janus particles (scale bar, 100 µm). **e** Scanning electron micrograph showing the vertical interface of o-SWNT/PTFE interface of the Janus particle (scale bar, 1 µm). **f** Top: Raman spectroscopic measurement of o-SWNT G band shift for CH_3_CN exposed o-SWNT (Raman 1) and polymer protected o-SWNT (Raman 2). Note a spectroscopic window on the polymer protected side was opened up to avoid polymer interference in Raman measurement. Scale bar, 100 µm. Bottom: Raman G band shift of o-SWNT before (red) and after (blue) CH_3_CN addition for the exposed (left, Raman 1) and polymer protected (right, Raman 2) side. **g** Top: schematic illustration of closed-circuit measurements to quantify the electrical output of such Janus particles, in which the particles are lowered into a reservoir of CH_3_CN, and the current and voltage profiles across a known external load are recorded. Bottom: Measured voltage profiles across a 5000 kΩ external resistor for particle generators of various sizes but identical aspect ratios (black or volume ×1, 500 µm × 250 µm × 250 µm; red or volume ×2, 636 µm × 315 µm × 315 µm, blue or volume ×3, 720 µm × 360 µm × 360 µm). **h** Top: schematic illustration of Janus particles of the same volume (2 mm^3^), but different aspect ratios (AR) of 0.5 (blue), 1 (red) and 2 (black). Bottom: Current–voltage characteristics of the three particles represented above. Error bars represent standard deviations of different measurements using different replica of devices (*n* = 3). Colored squares represent the maximum output power of devices at each AR if the external resistance is impedance-matched to the device internal resistance.

Recent advances in understanding molecular interactions with nanostructured materials have led to a myriad of energy harvesting schemes, coupling inputs of various kind to the direct generation of electricity, mediated by the solvent molecules adsorbed onto the nanostructures^1–6^. There have also been examples of electric energy conversion solely from the spontaneous adsorption and desorption of various solvents^7–12^. The asymmetric adsorption of water molecules across nanostructure protein thin films, for example, has been shown to produce continuous electrical power at 0.5 V and 17 µA∙cm^−2^ ^11^. Voltage outputs and power densities exceeding 1.3 V and 1.0 mW∙cm^−2^ have also been reported for water adsorption onto a graphene oxide device with a preformed axial oxygen gradient^8^. Follow-up studies extended such adsorption-based electricity generation into other solvent systems, like methanol (c.a. 30 mV) and acetone (c.a. 20 mV), providing opportunities for electricity generation in non-aqueous environments^10^. As a mechanism of solvent-induced electron flow, we have recently introduced asymmetric chemical doping (ACD), a chemical potential gradient of electrical carriers in nanostructured carbon materials, established using an asymmetric adsorption of acetonitrile (CH_3_CN) molecular dopants, as a mechanism for adsorption-induced electricity generation^13,14^. In this process, the broken spatial symmetry in the Fermi levels (*E*_F_) of electrical carriers inside a single-walled carbon nanotube (SWNT) network translates directly into a voltage potential. This “rechargeable” electric output (with specific power as large as 26.7 mW∙mg^−1^) stems from the electron transfer process between the SWNT donor and the p-doping CH_3_CN acceptors, and can be generalized into a large family of small molecule organic solvents^13,15^. With a tunable voltage output (up to 920 mV open-circuit potential), and a wide range of compatible solvents including the CH_3_CN, in this work we take advantage of this phenomenon in the design of a carbon Janus particle that generates solvent-induced electricity for electrochemical reactions (Fig. 1a). CH_3_CN is chosen for its small molecular size, appropriate energy level of molecular orbitals, and its suitability as solvent for various electrochemical reactions. Although Janus structures have been widely employed to propel micromotor particles by utilizing the energy from spontaneous chemical reactions^16–21^, our device represents the first example of converting solvent adsorption energy into electrical potential to power electrochemical reactions that would otherwise not proceed spontaneously. Recent developments in organic electrosynthesis such as the allylic C–H oxidation^22^ and the decarboxylative etherification^23^ provide opportunities for fine-chemicals industry to use electron current in place of stoichiometric chemical oxidants or reductants. In the present work, we fabricate symmetry-broken, carbon particles comprised of high surface area SWNTs and demonstrate their ability to convert exothermic solvent adsorption into usable electrical potential, turning over electrochemical redox processes in situ with no additional external power supplied. This particulate form factor for solvent-based electron flow has the advantage of facile integration into existing high-throughput analytical methods such as nuclear magnetic resonance (NMR) and UV-Vis for parallel screening of reaction products. The concept of solvent-generated electrical power coupled to the active surface of a catalytic particle represents a fundamentally new way of driving electrochemical reactions.

## Results and discussion

### Performance and mechanism of particulate generator

The particulate voltage generator (Fig. 1a) utilizes the mechanism of ACD^12,13^, in which a spatial asymmetry in exposed SWNT surface area to CH_3_CN is achieved by biasing the CH_3_CN solvent access toward one half of the SWNT particle (Fig. 1b). Here, the solvent p-dopes or withdraws electrons from the exposed carbon surface, creating a Fermi level gradient along the SWNT conductor between two electrochemically active surfaces, ultimately driving electron flow across the particle. To create particles capable of solvent-induced galvanic potential, we hot-pressed purified SWNT powder into 500-µm thick sheets, with one side coated with a barrier polymer material such as Nafion, polyvinylalcohol (PVA), or polytetrafluoroethylene (PTFE). Dicing these sheets into 250 µm cuboids creates carbon Janus particles (Fig. 1c–e), leaving only the exposed (unprotected) surface with direct access to the surrounding solvent. Profilometry shows flat cubic particles with RMS roughness of 23.3 µm, and Raman spectroscopy maps a spatially uniform SWNT Fermi level of −0.19 eV (consistent in an ambient O_2_ environment) with G band Raman shifts around 1589 ± 1 cm^−1^ (Fig. 1f). Upon insertion of the Janus SWNT/polymer particle into a reservoir of CH_3_CN solvent, this polymer layer serves as an transport barrier between the SWNTs from the bulk CH_3_CN, providing the spatial asymmetric CH_3_CN doping gradient in the axial direction of the underline SWNTs necessary for the induced electric potential (Fig. 1h). The resulting CH_3_CN-induced Fermi level gradient in SWNTs is evident spectroscopically as a corresponding Raman G band shift of 15 cm^−1^ between the CH_3_CN exposed and polymer protected side (Fig. 1f).

We immersed the pristine (or non-oxidized) SWNT/PTFE asymmetric particles into a series of p-doping (electron withdrawing) solvents (rank ordered by the energetics of their lowest unoccupied molecular orbital, or LUMO levels) at ambient conditions (one particle per 2 mL of solvent), and measured the resulting open-circuit voltage output with copper electrodes physically inserted into the two ends of the asymmetric carbon device. Control studies were performed to rule out the electrochemical presence of the copper electrode (see Supporting Information 3-1), and all subsequent electrochemical reaction experiments throughout this study were conducted in the absence of copper. We observed a characteristic Gaussian-shape dependence of the resulting open-circuit electrical potential as a function of the dopant LUMO level, in agreement with previous findings (see Supporting Information 3-2)^15^. In these devices consisting of pristine-SWNTs, fluoro-acetonitrile (F-CH_2_CN) appears to be the optimal solvent of choice for voltage generation (c.a. 440 mV), whereas CH_3_CN doping affords approximately 160 mV. Subsequent oxidations of the pristine-SWNTs, following a reported procedure^13^, enhanced the CH_3_CN-mediated voltage output monotonically, up to 920 mV (highest for devices fabricated using SWNTs with 25% surface oxygen atom coverage), owing primarily to the increased effective SWNT specific surface area (and hence the effective Fermi level drop across the Janus SWNT interface, see Supporting Information 3-3). The correlation between the SWNT oxidation level and voltage output (of the device) suggests the ability to tune to a desired voltage by dialing in a single material parameter.

Based on these results, we selected oxidized-SWNT (o-SWNT) ([O] = 25% surface oxygen atom coverage determined with X-ray photoelectron spectroscopy (XPS)) as the active component in the Janus o-SWNT/PTFE particles. We characterized these particles as closed circuits to quantify output power (Fig. 1g, top). Current and voltage were recorded upon particle immersion into a CH_3_CN reservoir, and the discharge voltage curves measured across a 5000 kΩ external load suggest a strong volume dependence of the charge capacity (Fig. 1g, bottom), with an estimated total charge of 0.039, 0.065, 0.094 µA∙h for particles of sizes 0.031, 0.063, and 0.093 mm^3^, respectively. The Janus particle immersed in the solution forms a dynamic junction, which continuously extracts energy from additional CH_3_CN adsorption events onto “dry” SWNTs protected by asymmetrically laminated polymer barrier as the CH_3_CN dopant molecules diffuse in. This is supported by our previous observation of a moving wavefront between the “doped/wetted” and “undoped/dry” SWNT as we soak the particles with CH_3_CN. Once the entire particle is wetted by CH_3_CN, the transient electricity generation stops. The duration of electricity generation is determined mainly by the speed of solvent diffusion, which is influenced by the wettability of SWNT (ex. oxidation level), internal pore size distribution, as well as device length^15^. This amounts to 1.45 nmol of electrons mobilized by the smallest particle tested, in principle capable of driving a 1.45 mM redox couple in a 1 µL CH_3_CN solution. It is also worth noting that these discharges occur approximately at the same voltage (c.a. 730 mV), regardless of particle volume, and remain stable for most of the discharging period. We further characterize device power outputs as a function of their aspect ratios (defined as the ratio between particle cross-sectional area and its axial length, perpendicular to the Janus faces, Fig. 1h, top). Various external loads were tested to map out the power curves of each particle (Fig. 1h, bottom), indicating a linear enhancement in power output with increased particle cross-sectional area (Fig. 1h, also see Supporting Information 3-4).

### Ferrocene oxidation and metal ion reduction

**Fig. 2:**
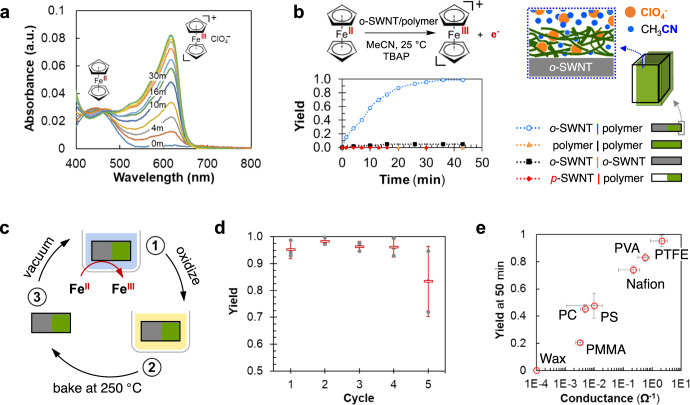
Ferrocene oxidation using in situ generated electricity. **a** Real-time UV-Vis absorption spectra of a well-stirred ferrocene (0.6 mM, in CH_3_CN) and tetrabutylammonium perchlorate (TABP, 50 mM, in CH_3_CN) solution, upon insertion of one o-SWNT/polymer particle (1 mm × 2 mm × 2 mm, AR = 4, volume = 4 mm^3^). The reactant (ferrocene) peak centers around 430 nm, whereas the product (ferrocenium perchlorate) peak centers around 615 nm. The UV-vis cuvette was filled with nitrogen to avoid interference from oxygen. **b** Top-left: reaction scheme of ferrocene oxidation driven by o-SWNT/PTFE particle generators. Bottom-left: control studies of the ferrocene oxidation kinetics using various particle constructs. Blue: standard condition, Janus particle made with o-SWNT half-coated with polymer thin film (100 nm); orange: o-SWNT particle fully coated with polymer; black: bare o-SWNT particle with no coating; red: Janus particle made with pristine non-oxidized SWNTs (p-SWNT) half-coated with polymer. All reactions are performed at 0.6 mM ferrocene concentration with 50 mM TBAP as electrolyte, with particle dimensions 1 mm × 2 mm × 2 mm. Right: schematic illustration of the cross-section of the 100 nm polymer coating. It also allows restricted solvent (blue dot)/electrolyte (orange dot) diffusion, as characterized by the solvent permeability through the polymer membrane. **c** The three-step Janus particle “recharging” cycle, consist of (i) re-oxidizing (using 2:1 volumetric mixture of concentrated HNO_3_/H_2_SO_4_) the reduced o-SWNT (during ferrocene electro-oxidation reaction at the exposed, anodic surface) on the cathodic (polymer protected) o-SWNT surface, (ii) rinsing off adsorbed chemicals on the particle and baking at 250 °C to get rid of rinsing solution and residual acids, and (iii) vacuuming away any residual liquid for the next reaction cycle. **d** Calibrated ferrocene yield with recycled Janus particles. Error bars represent 95% confidence intervals of oxidation yields using different particles (*n* = 3). **e** Calibrated ferrocene oxidation yield at 30 min of reaction time using o-SWNT Janus particles (1 mm × 2 mm × 2 mm) half-coated with different polymers (c.a. 100 nm). Device conductivities are measured between the two terminals of the Janus particles using infiltrated silver (Ag) nanoparticles (initially dissolved in CH_3_CN) as electrode contacts (see Supporting Information 10), which is a proxy for polymer permeabilities to CH_3_CN. Error bars represent the standard deviations of both reaction yield and conductivity measured within different samples (*n* = 3).

Quantitative conversion of ferrocene to ferrocenium ion can be achieved by dropping a single 1 mm × 2 mm × 2 mm o-SWNT/polymer Janus particle (AR = 4, volume = 4 mm^3^) into a well-stirred ferrocene/CH_3_CN solution (0.6 mM, 0.6 mL), using tetrabutylammonium perchlorate (TBAP, 50 mM) as an electrolyte, over a course of 30 min under ambient conditions (Fig. 2a). Similar reactivity is observed using 40 smaller particles (720 µm × 360 µm × 360 µm) with an equivalent effective total volume (see Supporting Information 4). Control studies using particles that have either no active generating ingredient (i.e., replacing o-SWNT to pristine-SWNT, with voltage output less than the reductive potential of ferrocene), or an unbroken symmetry (e.g., no Janus polymer coating), yielded negligible conversions (Fig. 2b), which rules out any non-electrically assisted chemical pathways. Also, because the ACD electricity can be repeatedly generated^13^, the Janus particles can be “recharged” by drying off CH_3_CN from the SWNT surface and re-oxidized in HNO_3_/H_2_SO_4_ mixture (2:1 volumetric ratio), allowing multiple cycles of ferrocene oxidation without significant degradations in yield (Fig. 2c, d). It should be noted that even though the electricity can be reversibly induced across the Janus SWNT interfaces from CH_3_CN adsorption, the use of the generated electrical potential for the productive electro-oxidative process (i.e., ferrocene oxidation) requires a corresponding reduction reaction on the cathodic side of the Janus particle, which needs to be replenished via the re-oxidation of the SWNTs. We emphasize, however, without the ACD-generated electrical potential, such two redox couples cannot occur spontaneously (Fig. 2b). In addition, the consumption of oxygenated functional groups would lower the generated voltage of the particles (Supporting Information 3-3), which is also amended during re-oxidation. Furthermore, we note that the oxidation kinetics is influenced by the mass transfer of the electrolyte and the redox pair (e.g., -COOH vs. -COO^–^ and H_2_) across the polymer membrane on the reducing (polymer protected) o-SWNT surface (i.e., the cathodic carbon surface, Fig. 2b, inset), which gives rise to the observed monotonic dependence of ferrocene oxidation yield on polymer permeability to CH_3_CN, a parameter re-casted in Fig. 2e as the measured electrical conductance. Detailed kinetic analyses reveal that the initial rate of the electrochemical process is limited by ferrocene adsorption on the anodic (exposed) o-SWNT surface (see Supporting Information 5). As a cautionary note, only a small fraction of charges mobilized from the ACD manage to successfully couple to the iron (II) oxidation reaction. We attribute this low electrochemical coupling efficiency (c.a. 0.2%) to a combination of capacitive and resistive losses between the particle electrodes and electrolyte. Nevertheless, this proof-of-concept experiment demonstrates the potential of exploiting the ACD electric current for chemical transformation, turning the adsorption enthalpy of organic solvent (i.e., CH_3_CN) on nanostructured carbon (i.e., o-SWNT) into an useful source of electric power, without the use of any complex, external circuitry.

**Fig. 3:**
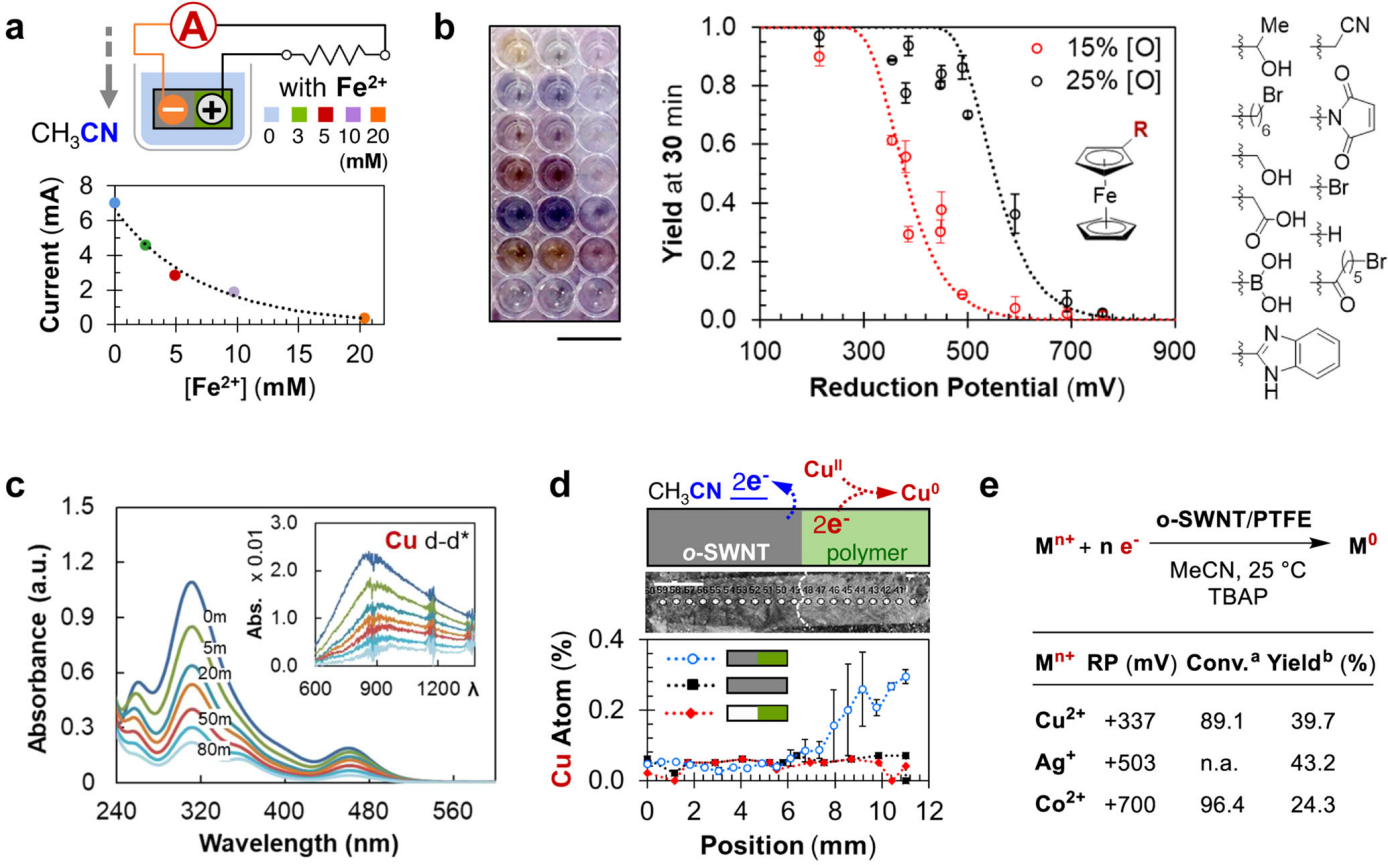
Electrochemical characterization using various redox pairs. **a** Top: schematic illustration of closed-circuit current measurements upon inserting a particle generator (1 mm × 2 mm × 2 mm, AR = 4, volume = 4 mm^3^) into various concentrations of ferrocene CH_3_CN solutions. **b** Left: optical image of 10 (in replica of two, so 20 total) parallel oxidation reactions using different ferrocene derivatives followed by a standard UV-Vis plate reader (scale bar, 1 cm). Right: calibrated yields of different ferrocene derivatives measured at 30 min of reaction time using o-SWNT/polymer particles at two SWNT oxidation levels (black: [O] = 25%, standard condition; red: [O] = 15%, as measured by XPS). These plate-reader-determined yields are ranked with the reduction potentials of these ferrocene derivatives, measured separately using cyclic voltammetry (see Supporting Information 8). Error bars represent standard deviations of the calibrated yields (*n* = 4). **c** Real-time UV-Vis absorption spectra of a well-stirred copper chloride (CuCl_2_, 0.6 mM, in CH_3_CN) and TBAP (50 mM, in CH_3_CN) solution, upon insertion of one o-SWNT/polymer particle (1 mm × 2 mm × 2 mm, AR = 4). Inset: the absorption peak of the d–d transition of the copper (II) complex (Cu^2+^). **d** Top: schematic illustration of the copper (II) reduction on the o-SWNT/polymer particle, showing the reduction is expected to take place on the polymer coating side. Middle: optical image of the 12 mm × 2 mm × 160 μm (AR = 0.27, volume = 4 mm^3^) o-SWNT/Nafion device used, as viewed from the XPS vacuum chamber. Each spot represents a sampling point for X-ray excitation, with the Janus interface indicated by the dotted line (scale bar, 2 mm). Bottom: calibrated metallic copper (Cu^0^) atomic percentage along the axial direction of the o-SWNT/polymer Janus particle (blue) after Cu^2+^ reduction reaction. Control particles of o-SWNT with no polymer coating (black) and Janus particles made with pristine-SWNTs (red) are also tested. Error bars represent standard deviation of XPS atomic ratio measurements using different samples prepared in separate reduction runs (*n* = 3). **e** Reaction scheme, electrochemical reduction potentials^27^, conversions, and yields estimated for various metal ions. ^a^The conversions are determined using UV-Vis absorption spectroscopy. ^b^The yields represent lower bounds as estimated from XPS elemental mappings (see Supporting Information 3).

To further explore the electron transfer between ferrocene and o-SWNT, we examined which side of the o-SWNT/polymer Janus particle the ferrocene oxidation occurs. This was probed by grafting a thin layer of poly-ferrocenylmethyl-methacrylate (PFMMA)^24^ over the entire particle surface and subjecting the PFMMA-grafted o-SWNT/polymer into a CH_3_CN/electrolyte solution. Since the only ferrocene molecules available for electrochemical oxidation are covalently linked to the PFMMA backbone, they are spatially fixed to the particle surface. Post-reaction analysis reveals that only the ferrocene molecules bound to the bare o-SWNT side were oxidized into ferrocenium, leaving those on the polymer protected side mostly in their original reduced state (see Supporting Information 6-3). This reaffirms that ferrocene exclusively oxidizes on the electron deficient, unprotected o-SWNT surface (i.e., the anode, Fig. 1a), driven by the ACD process that lowered the *E*_F_ on that side (Fig. 1b). Moreover, the electrochemical redox reaction consumes the ACD-generated electron flow, and necessarily reduces the solvent-powered closed-circuit current, which was also observed experimentally (Fig. 3a).

A particle that generates electricity with tunable charge capacity (i.e., through particle volume), voltage magnitude (i.e., through SWNT oxidation level), and power density (i.e., through aspect ratio), all of which is triggered by solvent interactions, provides an ideal platform for solution based electrochemistry. Furthermore, the ability to couple the internal electron flow reliably with a chemical process, especially one that results an appreciable shift in the absorption color spectrum (e.g., from Fe^2+^ to Fe^3+^), motivates a multiplexed reaction-detection scheme for high-throughput reaction monitoring, using standard UV-visible light plate readers. Here we subject ten ferrocene derivatives prepared with the same exact standard solution (except for modifications on the cyclopentadiene ring, Fig. 3b) to ten identical o-SWNT/PTFE particle generators tethered individually to ten wells (with replica of two) in a well plate (Fig. 3b, left). Subsequent parallel oxidation reactions occur within a plate reader that follows the product absorption peak for each derivative in real time. The calibrated yields (of the oxidized product, after 30 min reaction time, Fig. 3b, black circles) appear to be a function of the reduction potential (RP, see Supporting Information 8) of each derivative. With each particle generating approximately the same voltage, the ferrocene derivatives with lower RP (hence higher over potential, *η*) reacted faster, resulting in higher oxidation yields at 30 min. Moreover, we find that these results can be described by the Butler–Volmer equation (Fig. 3b, dotted lines), which couples the first-order oxidation kinetics to the particle anode current density:

1.1 $$[F{e}^{3+}]={[F{e}^{2+}]}_{0}-{[F{e}^{2+}]}_{0}\exp \left(-t\cdot {k}_{0}\exp \left\{\frac{(1-\alpha )zF\eta }{RT}\right\}\right)$$

Here [*Fe*^2+^]_0_ denotes the starting ferrocene concentration; *t* and *T* represent reaction time and system temperature; *k*_0_ and *α* are material parameters called intrinsic rate constant and charge transfer coefficient; *z* measures the number of electrons involved in the electrode reaction; *F* and *R* are Faraday and ideal gas constant, respectively. The model estimates that the o-SWNT/PTFE would generate 746 mV of equilibrium potential (Fig. 3b, black dotted line), as compared to the closed-circuit device voltage output of c.a. 730 mV across a 5000 kΩ external resistor (Fig. 1g). In contrast, a set of less oxidized Janus SWNT particles ([O] = 15%, known to generate c.a. 550 mV of electric potential, see Supporting Information 3-3) exhibit much lower reactivity (Fig. 3b, red circles), a result expected from Eq. 1.1, with a reduced particle equilibrium potential. This functional group tuned reactivity, subject to variations of the particle equilibrium potential (which is ultimately controlled by the oxidation levels of the SWNTs), demonstrated the excellent chemo-selectivity of the electrochemical approach. To illustrate, one can selectivity suppress the oxidative yield of ferrocenyl-succinimide (RP ≈ 490 mV) from 87 to 8%, without affecting much the reactivity of α-methylferrocenemethanol (RP ≈ 215 mV, with a yield drop from 97 to 90%) by simply switching to the less oxidized o-SWNT/PTFE system (from [O] = 25 to 15%, Fig. 3b).

The ACD-powered electrochemistry is not restricted to the Fe^2+^/Fe^3+^ redox pair, rather, it should, in principle, encompass an array of transformations that falls into the electrochemical window (defined by the o-SWNT/polymer particle), such as, the reduction of copper (II) chloride (Cu^2+^ → Cu^0^, RP ≈ 337 mV, Fig. 3c). Yield calculations for metal ion reductions, we note, appear to be less straight forward, primarily due to the lack of a spectroscopic signature from the reduction product, as well as product (metal) depositions onto the particle cathode (the polymer protected side, Fig. 3d). This undesired deposition, however, offers a means to locate where on the particle the reduction has occurred. Both XPS (Fig. 3d) and scanning electron microscopy–energy dispersive spectroscopy (SEM-EDS, see Supporting Information 9) elemental analyses revealed substantial Cu^0^ deposition localized only on the polymer protected cathode, once again validating the proposed ACD reaction mechanism (Fig. 1a). Using quantitative elemental mapping, a lower bound of the copper (II) reduction yield is measured at 39.7%, similar to other metal ions within the electrochemical window, such as cobalt and silver (Fig. 3e).

### In situ driven selective alcohol electro-oxidations

**Fig. 4:**
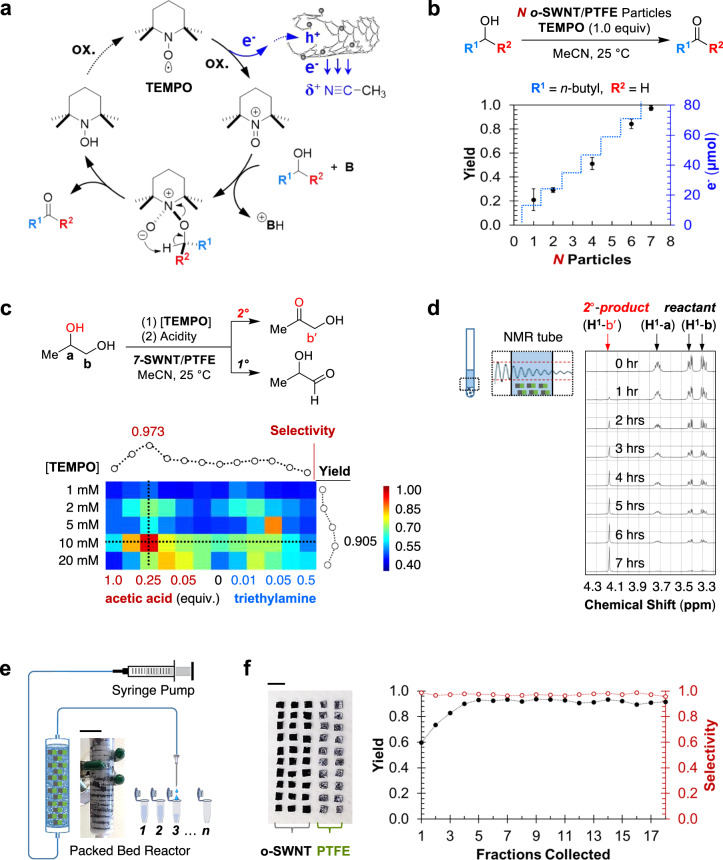
In situ driven selective alcohol electro-oxidations. **a** Proposed mechanism for o-SWNT/PTFE particle-enabled electrochemical partial oxidation of alcohols to ketones or aldehydes (e.g., R^2^ = H) in CH_3_CN, using TEMPO as a co-oxidant. **b** Calibrated yield for the partial oxidation of amyl alcohol to amyl aldehyde electrochemically using various numbers of o-SWNT/PTFE particles (1 mm × 2 mm × 2 mm). Reactions are conducted under ambient condition for 8 h at 20 mM reactant concentration. The blue dotted lines represent the total charge capacity of each scenario where *N* number of generator particles are used. Error bars represent standard deviations of reaction yields collected for each number of particles used (*n* = 3). **c** Selective oxidation of secondary (2°) over primary (1°) alcohol in 1, 2-propadiol. Top: reaction scheme of (1) TEMPO loading and (2) solvent acidity screen for the o-SWNT/PTFE-assisted electrochemical oxidation of 1, 2-propadiol, with both potential oxidation products listed. Protons (^1^H-a, ^1^H-b) of the reactant, as well as the proton (^1^H-b′) of the 2°-oxidation product (1-hydroxyl-2-propanone), are labeled accordingly. Bottom: selectivity toward the 2°-oxidation product, displayed both as a function of the TEMPO loading (vertical axis) and solvent Lewis acidity (horizontal axis, tuned via the addition of various equivalents of acetic acid—more acidic—or triethylamine—more basic). The yield along the vertical dotted line is also reported on the right hand side. **d** Top: schematic illustration of the in situ NMR kinetic measurement of 1, 2-propadiol oxidation using the conditions optimized in Fig. 3d. D_3_-acetonitrile (CD_3_CN) is used as the solvent. Bottom: assigned ^1^H-NMR spectra for the reaction mixture. **e** Schematic illustration of the packed bed electrochemical reactor (PBER), in which a plug flow reactor column is packed with 50 standard o-SWNT/PTFE particle electricity generators (1 mm × 2 mm × 2 mm), for multi-stage continuous electrochemical production of 1-hydroxyl-2-propanone. Fractions are collected with an automated system at 30 min per cut. A total of 18 fractions are collected for a 9-h run. Inset: the prototype PBER used in this study (scale bar: 1 cm). **f** Left: optical image of the 50 o-SWNT/PTFE particle generators used to construct the PBER, with either the o-SWNT side or the PTFE-coated side facing up (scale bar: 5 mm). Right: calibrated yield and selectivity for 1-hydroxyl-2-propanone in each fraction collected from the PBER.

The solvent CH_3_CN is used routinely as a non-aqueous electrochemical medium and also functions in this case as a dopant to power the ACD-driven electrochemistry. To further demonstrate the synthetic utility of these solvent-derived electricity, we turned our attention to an organic system with industrial relevance—the partial oxidation of alcohols to aldehydes or ketones. Selective oxidation of primary and secondary alcohols to the corresponding carbonyl compounds is a fundamental and important transformation in organic chemistry^25^, which is traditionally resolved by an electrochemical oxidation using a nitroxyl radical (2, 2, 6, 6-tetramethylpiperidin-1-yl)oxyl, or TEMPO, as a catalytic mediator (Fig. 4a)^26^. Here we hope to apply our ACD-generated electric potential to drive the desired partial oxidation in a stoichiometric fashion. To lower the detection burden on the analytical methods (i.e., NMR, spectroscopy), we increased the reactant concentration to 20 mM. This in turn meant that we had to adjust the number of the o-SWNT/PTFE particles accordingly. We observed essentially no desired product in the absence of either the Janus particles, the TEMPO co-oxidant, or the CH_3_CN, consistent with an ACD facilitated mechanism (Fig. 4a). A brief optimization on the number of participating Janus particles suggests that seven generators can fully account for the increase in carriers needed to drive the model amyl alcohol oxidation to completion (Fig. 4b). The linear scaling of the oxidative yield as a function of particle number shows that each provides a specific quanta of electrochemical energy. This method also proved to be compatible with a range of reactants. An array of primary (entry 1–8) and secondary (entry 9–12) alcohols can effectively couple to the oxidative cycle with good yield (Table 1).

**Table 1:** Yield for the electrochemical partial oxidation of various primary (entry 1–8) and secondary (entry 9–12) alcohols by o-SWNT/PTFE particles.

Entry	R^1^	R^2^	Yield^a^ (%)
1		H	89 (80^b^)
2		H	98 (87^b^)
3		H	71^b^
4		H	>99
5		H	99
6		H	99
7		H	98
8		H	97
9			90^b^
10			87^b^
11			85^b^
12			85^b^

In all cases, the standard condition shown in Fig. 3b, with *N* = 7, is applied.

^a^Yields are calibrated with gas chromatography (GC).

^b^For the cases where GC analysis proves to be difficult (e.g., due to molecular weight), proton nuclear magnetic resonance spectroscopy (^1^H-NMR) is used instead.

These Janus carbon particles can be used for in situ powered electro-oxidation as a high-throughput optimization tool, since the particles function as both the electron source and high surface area electrodes with the energy derived from the solvent–carbon interactions. As an example, we tethered these Janus particles to each of the wells in a standard 96-well plate as an array of electrochemical cells. This allows us to evaluate multiple reaction parameters in parallel. We tested this system on the selective oxidation of propan-1,2-diol (Fig. 4c, top). In order to optimize the selectivity toward the secondary (2°) alcohol over the primary (1°) one, two parameters—the concentration of the TEMPO mediator, and the solvent acidity—were modulated in combination to identify several selectivity hotspots (Fig. 4c, bottom). Both slightly acidic and basic conditions seemed to, surprisingly, favor the oxidation at the 2°-location. This observation is consistent with a Lewis acid–base protection scheme that kinetically traps the 1°-alcohol at the second step of the TEMPO catalytic cycle (Fig. 4a). The optimized condition (10 mM TEMPO, 5 mM acetic acid additive) selectively furnished the 2°-product (hydroxyacetone) with respectable yield (91%) and selectivity (97%).

As a consequence of the relatively small footprint of these Janus particle generators (e.g., 4 mm^3^), one can easily incorporate them into a NMR tube and other measurements that use this form factor for in situ electrochemical kinetic measurements (Fig. 4d, top). By sinking these particles below the active spectroscopic window, electrochemical transformations can be followed, in real time, without magnetic interferences. We monitored, for example, the hydroxyacetone electro-oxidation following the reaction conditions optimized above, in deuterated acetonitrile (CD_3_CN) as solvent (Fig. 4d, bottom).

The idea of dividing electricity into modular and customizable units enables development of advanced chemical reactor configurations for larger-scale reactions. As such, a packed bed electrochemical reactor (PBER) was built, by vertically stacking 50 of these 4 mm^3^ o-SWNT/PTFE generators, in between insulating filter stages, to prevent electric shorts (Fig. 4e, f, left). A syringe pump was used to supply a steady influx (0.8 mL∙h^−1^) of the reactant (1, 2-propadiol), as well as the prior optimized reagent composition, all dissolved in CD_3_CN, into the PBER for 9 h of continuous hydroxyacetone production (Fig. 4f, right).

In conclusion, catalytic Janus particles have been created that utilize ACD as an electron source from the ambient fluid to enable solvent-driven electrochemistry. Compressed SWNTs with a polymer barrier face can induce electric current across the particle from adsorption of the surrounding solvent such as acetonitrile. These Janus particles generate electrical energy based on the charge capacity throughout the particle volume and the SWNT oxidation levels driven by solvent interactions alone. Our study represents the first report that (1) converts the electricity harvested from solvent adsorption into driving potentials for electrochemical reactions (2) in the organic phase, (3) all within the form factor of microscopic particles that may open up other opportunities for researchers in the particle community. The concept of harvesting and dividing electricity into modular and customizable units and incorporating them as synthetic building blocks may create opportunities in using preparative-scale electrolysis for molecular assembly. Merging energy harvesting with on-demand electrochemistry via particulate devices extends artificial chemical processes into previously inaccessible locations.

## Methods

### Preparation of purified SWNT powder

SWNT powders were purchased and used directly in the present study (HiPCo, Nanointegris Technologies, Inc). Water-soluble impurities were removed three times by extraction via a water/hexane system. Then, catalyst residues were removed by hydrochloric acid purification. After complete grinding and drying process, purified SWNT powders were used to make samples, such as the asymmetric particles used in this work (see Supporting Information 1-1 and 1-2).

### Typical procedure of sample and device preparation

SWNT networks were prepared using a hot-press method using Laboratory Press (Carver, Inc. Model #3912). In a typical procedure, 30 mg of SWNT powder was put on the Teflon sheet and 50 μL of DI-water was added to ensure network rigidity, with another Teflon sheet covering the SWNT powder. Hot-press was done by 5 tons at 50 °C for 10 min. After the pressing, SWNT network was dried overnight, and subsequently diced into specific sizes. The diced SWTN network was placed across two copper electrodes and were fixed with copper tapes to make a device for voltage and current measurements. Polymer coating (PVA) on the copper electrodes was added in order to protect the connection between SWNTs and electrodes from non-aqueous liquid acceptors (see Supporting Information 1-4 and 1-5).

### Typical procedure of electrical measurements

To evaluate basic electrical properties of SWNT network, electrical contacts were made on both sides of network using copper electrodes. Then, the particles were immersed, with the electrodes connected to the voltage meter or current meter, into solvents while measuring the voltage or current output. For open-circuit voltage measurements, both electrodes were directly connected to the voltage meter (Yokogawa, Digital Oscilloscope DL1735E). As for the closed-circuit current measurements, the electrodes were connected to current meter (Keithley, Digital Multimeter 2002) and an impedance-matched external resistor is connected in series for voltage measurements across the external resistor simultaneously (See Supporting Information 3). Short-circuit current measurements were performed by directly connecting the current meter to the device without the impedance-matched external resistor (see Supporting Information 7).

### Typical procedure of asymmetric SWNT particle preparation

To create particles capable of solvent-induced galvanic potential, we hot-pressed purified and o-SWNT powder into 500-µm thick sheets, with one side covered with a barrier polymer material such as Nafion, PVA, or PTFE. Dicing these sheets into 250 µm cuboids creates carbon Janus particles, leaving only the exposed (unprotected) surface with direct access to the surrounding solvent (see Supporting Information 2).

### Typical procedure of CV (redox potential) measurements

Redox potentials of ferrocene derivatives were measured using cyclic voltammetry (CV). The CH_3_CN solution was prepared with adding 50 mM of TBAP as an electrolyte, and then 1 mM of each ferrocene derivative was added to prepare the solution for the CV measurement. The measurement was scanned at 50 mV/s, using a glassy carbon electrode and Ag/AgCl electrode for working and reference electrode, respectively (see Supporting Information 8).

### XPS characterizations

The oxidation levels of SWNT and metal atomic concentrations on both sides of o-SWNT and polymer-coated SWNT were determined using XPS (ULVAC-PHI, INC. PHI VersaProbe II) with a monochromated Al Kα source. The oxygen and the reduced metal salts content were calculated as an atomic percentage from the integration of O 2p peak, Cu 2p peak, Co 2p peak, and Ag 3d peak, respectively, in the high-resolution scans (see Supporting Information 3).

### SEM observations

The asymmetric SWNT samples were imaged using a Zeiss Merlin field emission SEM and elementally analyzed using an EDS (see Supporting Information 6).

## Supplementary information

Supplementary information

## Data Availability

The data that support the plots within this paper and other findings of this study are available from the corresponding author upon reasonable request.
