# Supplementary material for: Solvent-induced electrochemistry at an electrically asymmetric carbon Janus particle
Source: Nat Commun. 2021 Jun 7;12:3415. doi: 10.1038/s41467-021-23038-7 (PMC8184849; doi:10.1038/s41467-021-23038-7)
Supplement: Supplementary file 1 — Supplementary information [file 41467_2021_23038_MOESM1_ESM.pdf]

Supporting Information for

Solvent-Induced Electrochemistry at an Electrically Asymmetric Carbon Janus Particle

Albert Tianxiang Liu,[†] Yuichiro Kunai,[†] Anton L. Cottrill, Amir Kaplan, Ge Zhang, Hyunah Kim, Rafid S. Mollah, Yannick L. Eatmon, Michael S. Strano*

* Corresponding author's email: strano@mit.edu

[†] These authors contributed equally to this work.

Table of Contents

1. Asymmetric Chemical Doping generators for electrical characterizations 2

1-1. Raw materials 2

1-2. Purification of SWNT powder 2

1-3. Characterization of SWNT powder 3

1-4. Preparation of SWNT network 5

1-5. Preparation of devices 5

2. Fabrication procedure of asymmetric SWNT particles 6

3. Electricity generating mechanism and characterizations 7

3-1. Transport model on Asymmetric Chemical Doping voltage generation 7

3-2. Electrical characterizations of SWNT devices using different doping solvents 12

3-3. Electrical characterizations of SWNT devices at different oxidation level using CH_3CN as solvent dopant 14

3-4. Electrical characterizations of asymmetric o-SWNT devices at different aspect ratios 18

3-5. Electron transfer model on Asymmetric Chemical Doping voltage generation 20

4. Validation of equivalency between large and small particles on the experiment of Ferrocene oxidation 31

5. Kinetics and rate limiting step analysis 32

5-1. Assessment of the role of o-SWNT surface catalysis 32

5-2. Derivation of rate laws for three distinct limiting cases (adsorption, reaction, or desorption) 34

5-3. Experimental validation of rate law of ferrocene electro-oxidation 38

6. Ferrocene-polymer oxidization details 39

6-1. Polymerization 39
6-2. Polymer coating on SWNTs network 40
6-3. Characterization of PFMMA oxidation state 40
7. Keythley measurement with changing ferrocene concentration 46
8. CV(redox potential) of ferrocene derivatives 48
9. XPS and SEM of Copper, Cobalt, Ag reduction 49
9-1. Polymer coating on SWNTs network 49
9-2. Reaction 49
9-3. XPS analysis 49
9-4. SEM analysis 50
10. Procedure of resistance measurement of polymer-coated SWNT 53
11. Hypothesis on mechanism of long-lasting voltage creation in the reaction solution. 55

1. Asymmetric Chemical Doping generators for electrical characterizations

1-1. Raw materials

SWNT powders were purchased (HiPCo, Nanointegris Technologies, Inc).

1-2. Purification of SWNT powder

Further purifications were done using an extraction method to remove a water-soluble impurity and an acid purification method to decrease a catalyst residue. Firstly, the extraction of water-soluble impurity is done using hexane/water system. After SWNT powder is dispersed in pure water by bath sonication, hexane is added to water/SWNTs solution, then it is shaken for 1 minute to extract all the SWNTs from water phase to hexane phase. After the water phase is disposed, fresh pure water is added again and shaken to extract the water-soluble impurity more. This process

is done 3 times, and then SWNT powder is collected from hexane phase using glass filter with reduced pressure. SWNT powder is ground and dried on the glass filter for 15 minutes, then transferred into a vial and dried further for overnight under high vacuum condition. Secondly, acid purification is done using non-oxidative 37% hydrochloric acid (Aldrich). After adding the hydrochloric acid to SWNTs powder, the solution is bath-sonicated for 15 min to disperse SWNT powder. Then, solution is kept at 45°C for 2 hours with stirring. The hydrochloric acid solution is diluted with pure water and SWNTs is collected using glass filter with reduced pressure. The SWNT powder is washed by pure water 10 times and by acetonitrile once, then ground and dried for 15min on the glass filter. Grinding process is important to make a uniform SWNT network by hot-press method. The powder is transferred into a vial and dried further for overnight under high vacuum condition.

1-3. Characterization of SWNT powder

Purified powder samples were examined by XPS, BET, and UV-VIS-NIR for a characterization. In order to confirm the non-oxidation purification process, oxygen atomic concentrations on both pristine and purified powder samples were checked using XPS (ULVAC-PHI, INC. PHI VersaProbe II) with a monochromated Al Kα source. The oxygen content value was calculated as an atomic percentage from the integration of O1s peak and that of C1s peak in the high-resolution scans as shown in **Table S1-1**. It was confirmed that there was no obvious increase of oxygen content due to the oxidation during this purification process. Chlorine and Iron contents were also checked by XPS as shown in **Table S1-1**.

Table S1-1. XPS summary of SWNT powder

	pristine (atom%)	purified (atom%)
Oxygen	4.72	4.08
Chlorine	0.2	0.3
Iron	0.8	0.7

BET measurements with N_2 was done using an Accelerated Surface Area and Porosimetry System from Micromeritics (ASAP 2020). Samples are degassed at 150 °C, then surface area normalized by weight was measured at -196°C. Results are shown in **Table S1-2**. SWNT powder used in this study showed more than 500m^2/g BET surface area which typically well purified SWNTs have, so successful purification was confirmed by this BET measurement.

Table S1-2. BET result for purified SWNT powders

		HiPCo
BET surface area	m²/g	670.3
Langmuir surface area	m²/g	913.5
BJH adsorption surface area	m²/g	360.4
BJH desorption surface area	m²/g	412.9
average pore size	nm	9.0

UV-VIS-NIR chirality characterization was also performed. Initially, SWNT sample was dispersed in 2 wt% SDS solution for 20 hours using tip sonicator. Then, SWNT solution was centrifuged at 32000 rpm for 4 hours. Only supernatant was measured by UV-VIS-NIR scanning spectrophotometer (Shimadzu, UV-3101PC). After taking a spectrum, baseline was subtracted from the spectrum using the method of Kevin and Rishabh et al.[1] Spectra after subtraction were shown in **Figure S1-1**. Peak deconvolution of each chirality were also done to calculate the

chirality information by Lorentzians peak fitting using the method of Kevin and Rishabh et al.[1]

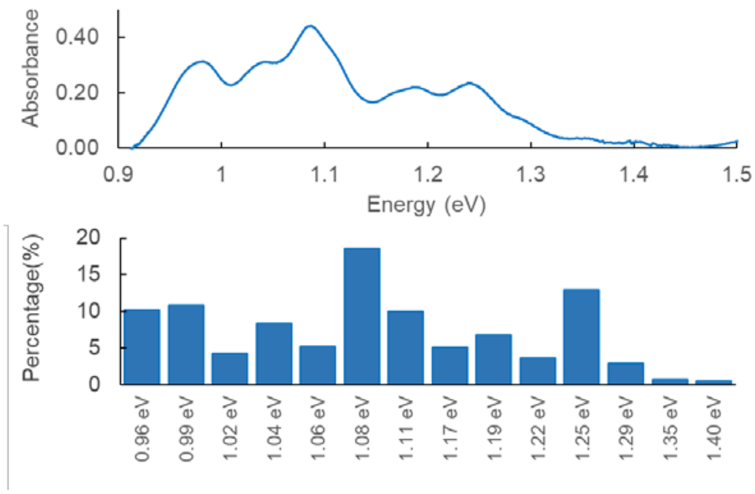

Figure S1-1. UV-VIS-NIR spectra in 2wt% SDS solution of HiPCo SWNT powder.

1-4. Preparation of SWNT network

SWNTs network was made by hot-press method using Laboratory Press (Carver, Inc. Model #3912). 30 mg of SWNT powder was put on the Teflon sheet and 50μL of water was added to make the network rigid, then another Teflon sheet covers the SWNT powder. Hot-press was done by 5 tons at 50 °C for 10 min. After the pressing, SWNT network was dried for more than overnight, then it was cut into specific sizes.

1-5. Preparation of devices

The cut SWTNs network was placed on across two copper electrodes then were fixed with another copper to make a device for voltage and current measurement. Polymer coating (polyvinylalcohol) on the copper electrodes were added in order to protect the connection between SWNTs and electrodes from liquid acceptors.

2. Fabrication procedure of asymmetric SWNT particles

SWNT particles were prepared using the method below, shown in **Figure 2-1**. To create particles capable of solvent-induced galvanic potential, we hot-pressed purified and oxidized SWNT powder into 500 µm thick sheets, with one side covered with a barrier polymer material such as Nafion, polyvinylalcohol (PVA), or polytetrafluoroethylene (PTFE). Dicing these sheets into 250 µm cuboids creates carbon Janus particles, leaving only the exposed (unprotected) surface with direct access to the surrounding solvent. If drying is performed on the particle preserved in solvent before actual use, then the shelf life should be months if not years.

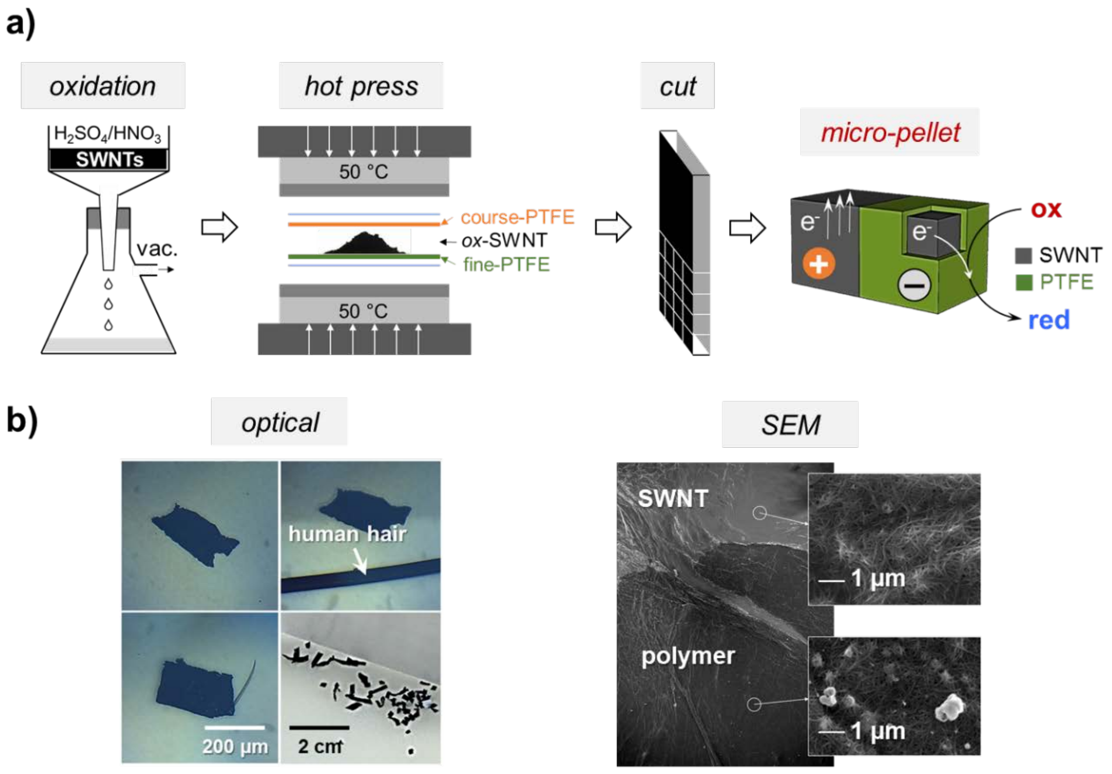

Figure S2-1. **(a)** The procedure of making o-SWNT particles used in this study, by oxidation and hot-press method, **(b)** Optical and SEM images of the particles.

3. Electricity generating mechanism and characterizations

3-1. Transport model on Asymmetric Chemical Doping voltage generation

The interaction between metals and CNTs in the side-contact geometry is determined by the hybridization between the carbon p_z orbitals and the unbonded orbitals of the metals. Alkali and simple metals have binding energy around 1.5 eV per atom. Some transition metal atoms with unpaired d electrons, such as Sc, Ti, Co, Ni, Pd, Pt, form strong bonds with a binding energy around 2.0 eV per atom, whereas the transition metals with fully occupied d orbitals such as Cu have relatively weak binding with a binding energy less than 1.0 eV per atom. The wettability of metals on CNTs is critical to the electrical transport properties at CNT-metal contacts. The correlation between the wettability of these metals and their binding energies on CNTs is clear, i.e., metals with relatively strong binding energies with CNTs tend to form uniform coatings.

The interaction energy between metal and SWNT (overlapping wavefunctions between electrode and SWNT) plays an important role in the probability of carrier transfer across the electrode/SWNT junction. Specifically, the overlapping integral (between the metal's and SWNT's wavefunctions) grows exponentially as interaction energy ($E_{binding}$) increases:

$$P_{CT} \propto A = k \cdot \left(\exp\left(\frac{E_{binding}}{k_B T} \cdot \alpha \right) - 1 \right) \qquad (0.1)$$

Here P_{CT} denotes the probability of carrier transfer across the metal-SWNT contact; A represents the overlapping integral between the two wavefunctions; k and α are scaling parameters.

In the case of Asymmetric Chemical Doping, upon lattice withdrawal of electron from the dopant exposed side of the SWNT, the desired carrier transport process is the drift-diffusion from the

undoped side to the doped side of the SWNT. This is a productive process that will increase the observed voltage output. However, simultaneous carrier transport from the metal electrode into the doped SWNT occurs as well, diminishing the observed voltage. The rate of carrier transport of this undesired process depends on energy gap (between the metal SWNT work functions) and the overlapping states (in a probabilistic sense) (**Fig. S3-1**).

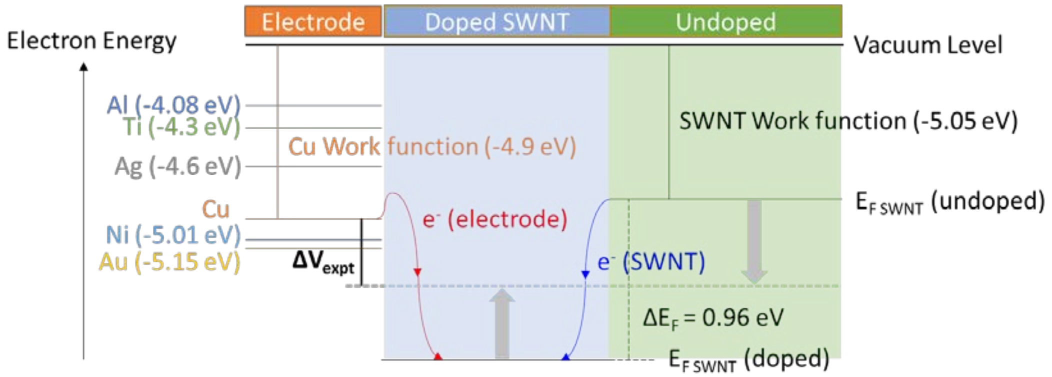

Figure S3-1. Electron energy diagram illustrating the desired (blue) and undesired carrier transport process in Asymmetric Chemical Doping. Different metal electrodes with different work functions are illustrated.

The combined rate of carrier accumulation within doped SWNT can be expressed as:

$$\frac{dN^{doped}}{dt} = k\left(\exp\left(-\frac{E_F^{doped}-E_F^{elec}}{k_B T}\cdot\beta\right)-1\right)\cdot\left(\exp\left(\frac{E_{binding}}{k_B T}\cdot\alpha\right)-1\right) + \frac{eF\eta}{L}\left(E_F^{undoped}-E_F^{doped}\right)+\frac{D}{L}\left(N^{undoped}-N^{doped}\right) \tag{0.2}$$

where N^{doped} is the carrier concentration on the side of the doped SWNT; E_F denotes the Fermi level; e is the elementary charge; F is Faraday's constant; η is carrier mobility; L is SWNT conduit length; and D is carrier diffusivity; k, α, and β are left as fitting parameters. Note that both the work function of the metal (E_F^{elec}) and its binding energy with the SWNT ($E_{binding}$) contributes to the rate of carrier transport across the metal-SWNT junction, with the work function affecting the

barrier height for the activated carrier transport process and the binding energy affecting the probability of that process. With this model, we can simulate carrier dynamics within the SWNT. To relate back to voltage output, we calculate the number density of electrons for (*n*, *m*)-SWNT, $N_e^{(n,m)}$, needed to lower the SWNT's Fermi level by 1 meV upon removal:

$$N_e^{(n,m)} = N_h^{(n,m)} - N_i^{(n,m)} = N_i^{(n,m)} \exp\left(\frac{\Delta E}{k_B T}\right) - N_i^{(n,m)} \quad (0.3)$$

Here k_B is the Boltzmann constant and T is system temperature, which remains constant at 298 K. ΔE represents the energy difference between the original and the reduced Fermi levels of the SWNT due to ET, which is set to 1 meV by definition. $N_i^{(n,m)}$ and $N_h^{(n,m)}$ represent the corresponding density of holes before and after the Fermi level reduction in the (*n*, *m*)-SWNT valance band (VB), respectively. $N_i^{(n,m)}$ is a strong function of the density of state (DOS) of (*n*, *m*)-SWNT, and is estimated following a procedure by Marulanda *et. al.*[2]

From our simulation results, we observe that the voltage output in ACD experiments is critically affected by $E_{binding}$ (**Fig. S3-2**). In both cases depicted in **Figure S3-2**, the metal electrode has a work function of 4.9 eV. On the left we simulated a scenario where $E_{binding}$ is 0.6 eV, whereas on the right we simulated the case when $E_{binding}$ is increased to 1.4 eV. We see that the resulting measurable voltage in both cases are 600 mV and 10 mV respectively (defined here as the difference between the terminal Fermi energy of the doped SWNT and the metal work function).

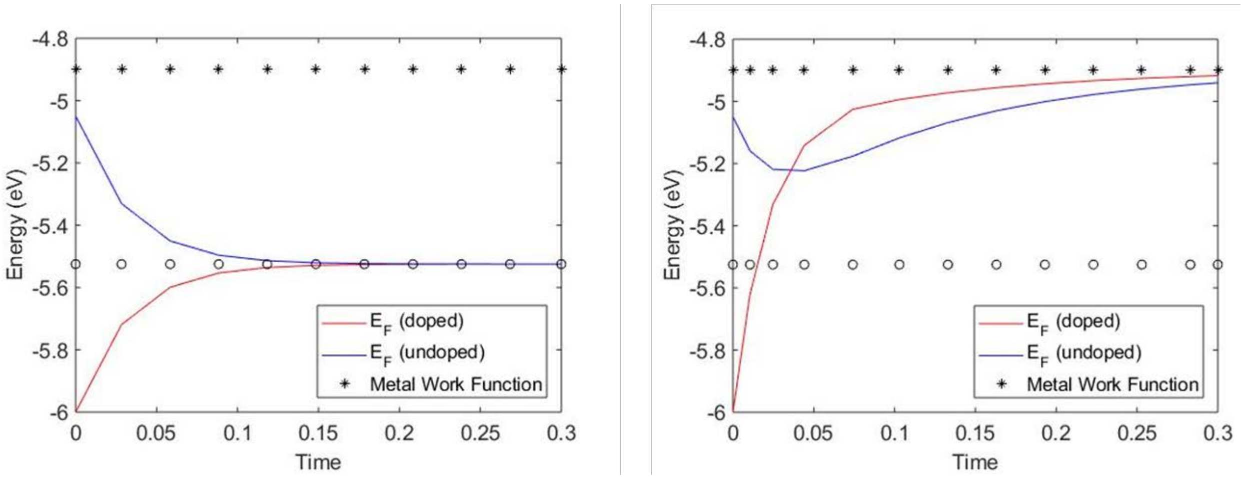

Figure S3-2. Simulated ACD Fermi level change for both doped and undoped SWNT as a function of time. On the left we simulated a scenario where $E_{binding}$ is 0.6 eV, whereas on the right we simulated the case when $E_{binding}$ is increased to 1.4 eV. In both simulation the metal work function is set to be 4.9 eV.

Using this model, we surveyed a wide range of $E_{binding}$ values and plotted the simulated short circuit current output as a function of these values (**Fig. S3-3a**) to highlight this dependence of voltage (and hence current) output as a function of $E_{binding}$ between metal and SWNT. A literature survey was also performed to estimate the binding energy between SWNT and different metal electrodes (**Fig. S3-3b**).

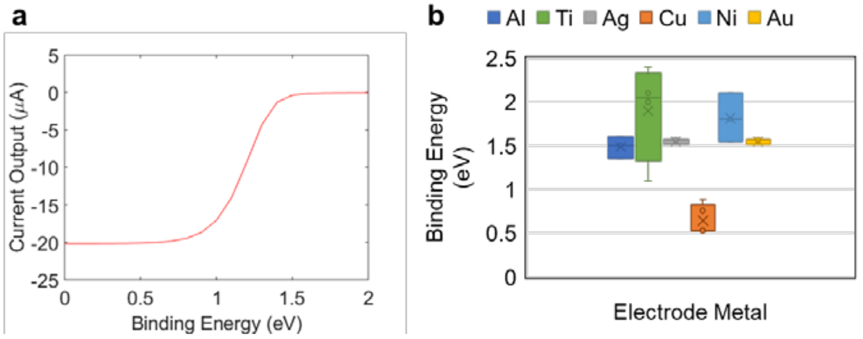

Figure S3-3. (a) Simulated short circuit current output as a function of the metal-SWNT binding energy. (b) Literature reported DFT calculated binding energy for various metal-CNT contacts.

We fabricated ACD devices using HiPCO SWNT with various metal electrodes (Al, Ti, Ag, Cu, Ni, Au) and experimentally measured the closed-circuit current outputs (R_{ext} = 1 kΩ) for these

devices (**Figure S3-4a**). In order to exclude potential galvanic contribution to the observed current, we did a control study in which the contact between SWNT and the electrode metal were cut off (**Figure S3-4b**). It is confirmed (from the equivalent circuit analysis (**Fig. S3-5**) that the galvanic effect contributes to less than 10% of the measured ACD current output.

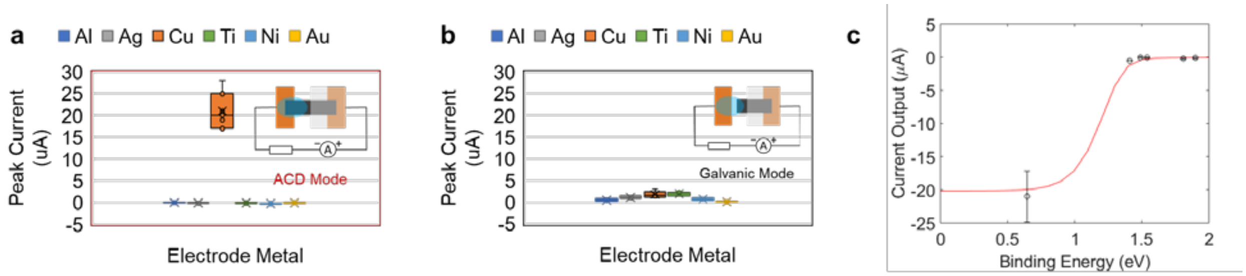

Figure S3-4. (a) Closed-circuit current measured for ACD devices fabricated using different metal electrodes (Al, Ag, Cu, Ti, Ni, Au). (b) Closed-circuit current measured for galvanic devices fabricated using different metal electrodes (Al, Ag, Cu, Ti, Ni, Au). (c) Overlay of the experimental result over model prediction.

Importantly, once we overlay the experimental results (**Fig. S3-4c**, black circles) on top of the predicted ACD current output (**Fig. S3-4c**, red line) using the aforementioned model, the quantitative agreement between the results and the model prediction confirms the important role binding energies between SWNT and the electrode metal play in the measurement of the ACD current (and voltage).

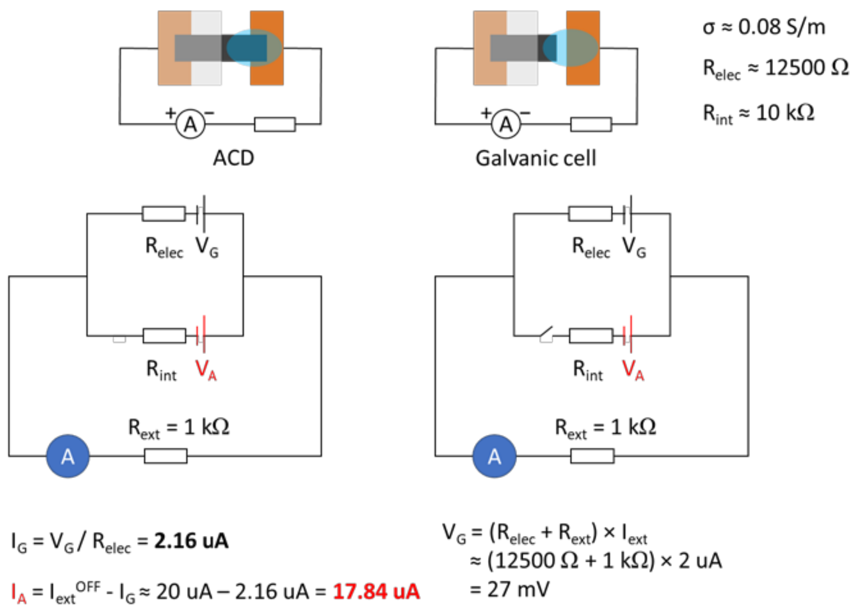

Figure S3-5. Equivalent circuit analysis for ACD and galvanic experimental set-up.

3-2. Electrical characterizations of SWNT devices using different doping solvents

Previous findings[3] have indicated that the open circuit voltage reached (as dictated by initial ratre of voltage generation or charge accumulation) by asymmetrically doping a hot-pressed HiPCO SWNT network using polar aprotic organic solvents follow a convoluted Gaussian curve as a function of the solvents' lowest unoccupied molecular orbitals (LUMOs). This was explained as an electron transfer limited process, following a modified Marcus electron transfer model (see Section 3-5 for more details). In addition, the liquid should be a good solvent for the reaction that will be powered by the Janus particle. The dopant molecule has to be small enough to enter the inter-tube spaces in SWNT bundles. Overall, acetonitrile (CH_3CN) is an appropriate solvent that satisfies all requirements well, and was hence chosen in our study.

Since we used hot-pressed HiPCO SWNT networks following a previously reported procedure[3] to fabricate the Janus polymer coated SWNT particles in the present study, these particles were tested

in a selected group of common organic solvents (selected from the same reported study) for voltage production in order to assess their abilities to produce self-generated voltage for electrochemical transformations when immersed in these solvent environments. We measured open circuit output to access the maximal electrical potential (and hence estimate the over potential available for electrochemical reactions) available in these devices, closed circuit measurements are also performed for a subset of the configurations tested, as shown in the main text and subsequent sections.

Using pristine (unoxidized, 10 % oxygen atom concentration by XPS characterization, see Section 3-3 for details) HiPCO SWNTs and a Janus polyvinyl alcohol (PVA) coating, we performed the study as schematically shown in **Figure S3-6a** (inset) with copper electrodes, immersing the SWNT/PVA particles into different solvents while measuring the voltage output. The open circuit voltage can be observed right after particle immersion and stabilize quickly to a maximum value (**Fig. S3-6b**). These maximum values are then plotted against as a function of the DFT calculated LUMO of these organic solvents. The dependence of the open circuit voltage as a function of the solvent LUMO can be described by a Marcus type electron transfer model reported previously.[3]

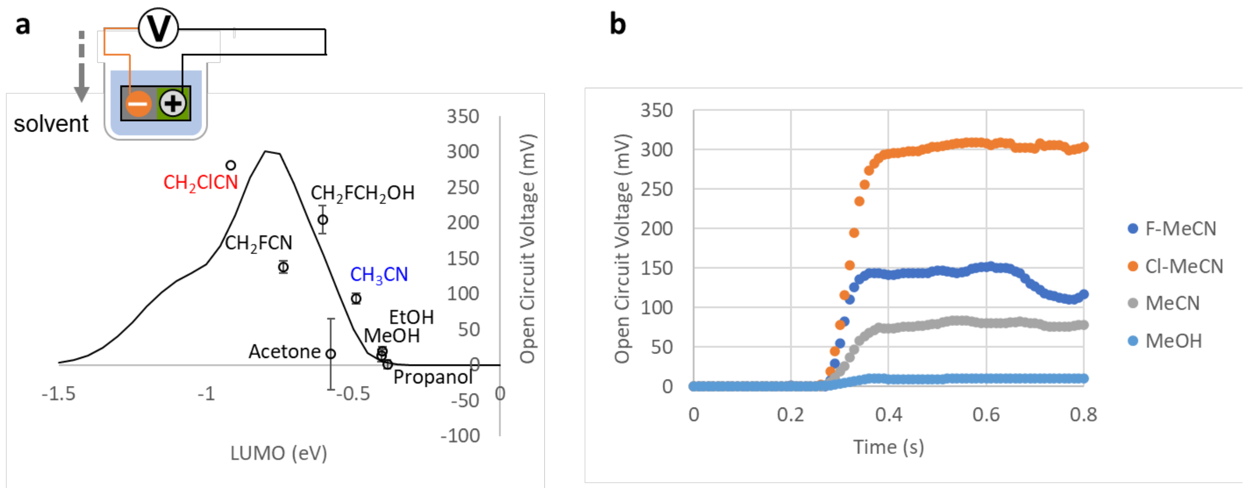

Figure S3-6. (a) Plots of the maximum open circuit voltage (open circles) on HiPCo SWNT as a function of the LUMO of molecular acceptors. The solvents studied here are (from low to high LUMO energy): chloroacetonitrile, fluoroacetonitrile, 2-bromoethanol, acetone, acetonitrile, methanol, ethanol, and propanol. The experiment is performed with PVA polymer coating, different from the PTFE coating used in the main text. The error bars represent 95% confidence intervals. LUMO levels were obtained by DFT calculation[3] on B3LYP/6-31G (d, p) level. The solid line indicates a fitting line based on a Marcus type electron transfer kinetics model[3] (see Section 3-4 for details). Inset: schematic illustration of the experimental set up for the open circuit voltage experiment. (b) Typical open circuit voltage profiles of HiPCo SWNT at early times of voltage generation for chloroacetonitrile (CH_2ClCN), acetonitrile (CH_3CN), and methanol (MeOH).

3-3. Electrical characterizations of SWNT devices at different oxidation level using CH_3CN as solvent dopant

Voltage output through CH_3CN doping on SWNT/polymer particle devices was tuned by the oxidation level of SWNT used for particle fabrication, experimentally tuned using acid treatment and pyrolysis. The acid treatment can increase the oxygen moieties and pyrolysis can remove them from SWNT surface. It is in principle possible to multiply the voltage of the particles by connecting them in series. However, this might have to be done by connecting several particles in separate solvent baths through external circuits, just like connecting enclosed batteries in series. We have preliminary data suggesting this is indeed possible, which we will reserve as the focus for follow up studies. For this study, the ability to tune the generated voltage of individual particles by varying the oxygen content is very convenient.

Acid treatment was done by soaking the SWNT powder into the mixture of sulfuric acid / nitric acid. We controlled the degree of modification using the concentration of acid solution. Concentrated acid solution (3:1 (v/v) of H_2SO_4/HNO_3) and diluted acid solution (3:1:40 (v/v) of $H_2SO_4/HNO_3/H_2O$) were used, for example. The acid-treated powders (after 20 s of acid treatment) were then washed with H_2O under stirring, and then with CH_3CN for two times under vacuum

filtration, then dried in the fume food under vacuum. The powders were then baked to 250 °C for 5 minutes on a hot plate (to ensure the removal any residue acids (in its free form), note both H_2SO_4 and HNH_3 evaporates long before it reaches 250 °C).

Pyrolysis was done under nitrogen atmosphere in the furnace of quartz tube. Samples was kept at 200 °C for 30min, then at 600 °C for 30 min, and finally at 1000 °C for 30 minutes. After baking the samples, we cooled it down under nitrogen atmosphere until it went back to room temperature. Then samples were transferred into closed vials which were filled with nitrogen gas in order to prevent re-oxidation.

Oxygen atomic concentration was investigated by XPS (ULVAC-PHI, INC. PHI VersaProbe II) with a monochromated Al Kα source. The survey scans of samples with different oxygen content. By the techniques of the acid treatment and pyrolysis, the oxygen content can be changed effectively from 4% to 27%. The oxygen content value was calculated as an atomic percentage from the integration of O1s peak and that of C1s peak in the high-resolution narrow scans in XPS (**Fig. S3-7**).

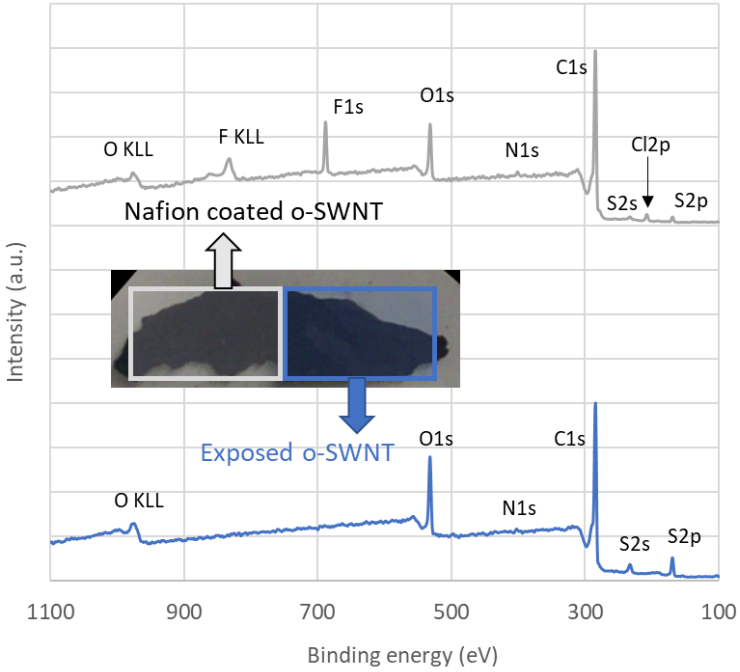

Figure S3-7. Example XPS scans of o-SWNT/Nafion Janus particle. Top spectrum is for the Nafion coated half and the bottom spectra is for exposed bare oxidized SWNT. Inset is an optical image of the sample taken prior to the XPS scans.

For the exposed oxidized SWNT side of the particles, we also found a linear correlation between the oxidation level of the o-SWNT with its sulfur atom content using XPS (**Fig. S3-8b**), but not with nitrogen atom concentration (**Fig. S3-8a**), indicating that the oxidized SWNT could bare functional groups (covalently bonded with SWNT carbon) such as sulfonates ($R\text{-}SO_3$), etc.

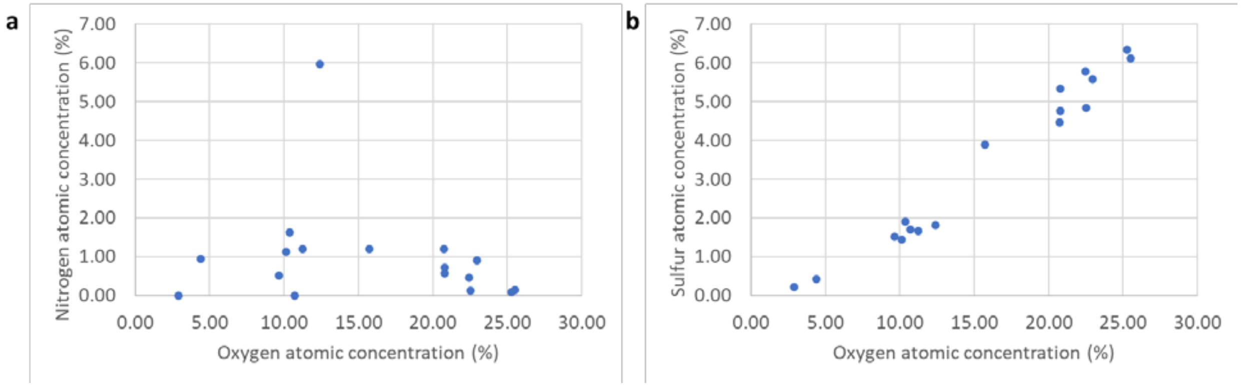

Figure S3-8. XPS determined nitrogen (a) and sulfur (b) atom percentage plotted against oxygen atom percentage for exposed (bare) o-SWNT.

We also found a linear correlation between the oxidation level of the SWNT with its sulfur atom content using XPS (**Fig. S3-9b**), but not with nitrogen atom concentration (**Fig. S3-9a**), indicating that the oxidized SWNT could bare functional groups (covalently bonded with SWNT carbon) such as sulfonates (R-SO_3), etc.

In terms of the open circuit voltage output of these devices, we noticed a super-linear dependence of maximum voltage reached by the asymmetric device (when inserted into CH_3CN), as shown in Figure S3-3.3a. This trend was observed previously, and the reason for such increased voltage potential was attributed to the increased surface area accessible to SWNT for CH_3CN adsorption. The correlation between the SWNT oxidation level and voltage output (of the device) highlights the ability to tune to a desired voltage by dialing in a single material parameter (oxygen atom content).

We have found that the voltage creation comes from the gap of Fermi-level between doped and undoped sides. If the surface area at specific volume is larger, more solvent molecules can deprive more electrons from CNT (more electron is withdrawn per carbon in the CNT lattice), this in turn creates a larger Fermi-level gap and a larger voltage.

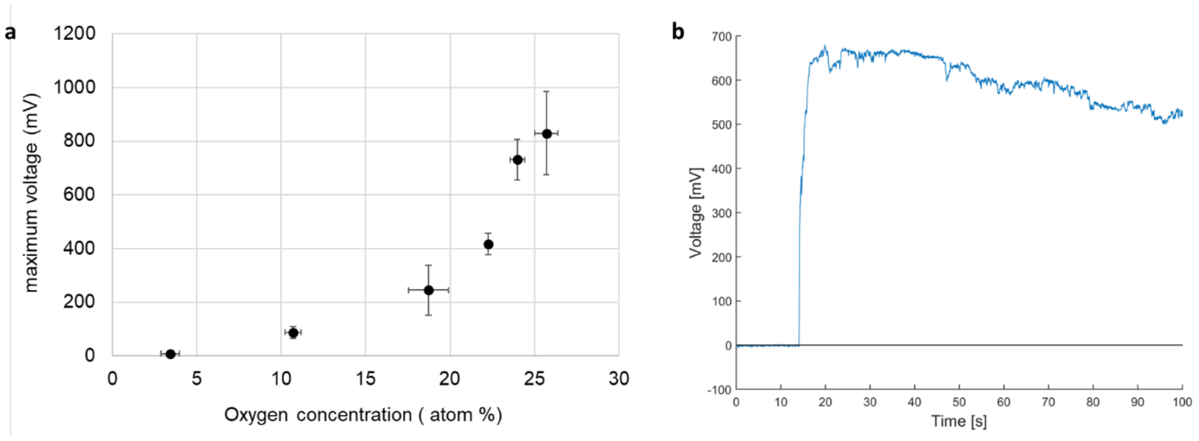

Figure S3-9. (a) Maximum CH_3CN doping voltage plotted against SWNT surface oxygen atom percentage determined via XPS. (b) Example voltage profile for an asymmetric device fabricated using 23.74 % [O] oxidized SWNT.

3-4. Electrical characterizations of asymmetric o-SWNT devices at different aspect ratios

We characterize device power outputs as a function of their aspect ratios (defined as the ratio between particle cross-sectional area and its axial length, perpendicular to the Janus faces, **Fig. 3-10b**). During the fabrication of such Janus o-SWNT particles, individual SWNTs are physically compressed into solid sheets with chosen thickness, as well as packing density and then, diced into rectangular particles with a laser beam (**Fig. 3-10a**), depending on the desired size and form factors. Various external loads are tested to map out the power curves of each device (**Fig. 3-10c**), indicating a linear enhancement in power output with increased particle cross-sectional area (**Fig. 3-10c**).

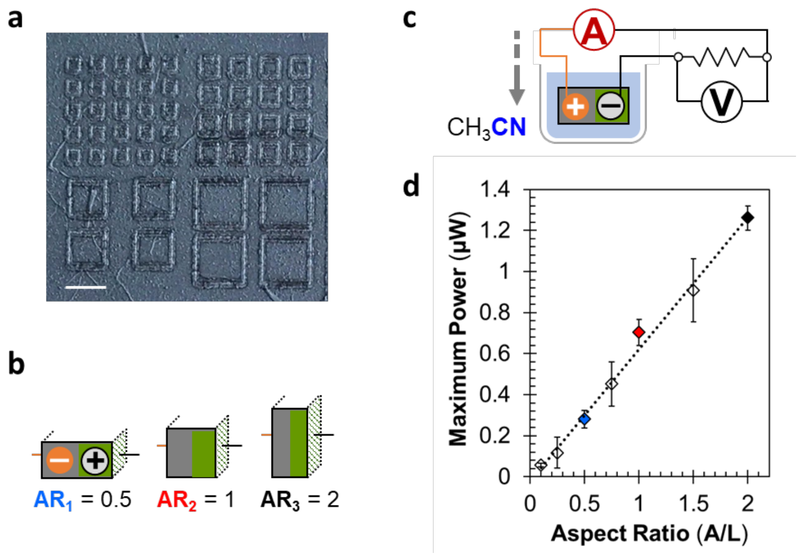

Figure S3-10. (a) Optical images of laser-cut edges on pressed o-SWNT flakes of different sizes (scale bar, 500 μm). (b) Schematic illustration of Janus particles of the same volume (2 mm^3), but different aspect ratios (AR) of 0.5 (blue), 1 (red) and 2 (black). (c) Schematic illustration of closed circuit measurements to quantify the electrical output of such Janus particles, in which the particles are lowered into a reservoir of CH_3CN, and the current and voltage profiles across a known external load are recorded. (d) Maximum output power plotted against Janus particle ARs. Error bars represent standard deviations of output powers measured using different devices (n = 3).

The external resistance used in these closed circuit measurements are: 5000 kΩ, 2000 kΩ, 500 kΩ, 200 kΩ, 100 kΩ, and 10 kΩ, and voltage signals across the external resistors are measured as a function of time (**Fig. S3-11**), where the peak voltages are used for power calculation. In all measurements, the voltage generation persists over several minutes. Note since the device internal resistance scales inversely with its aspect ratio (given the same device volume), the linear scaling of the maximum device power output as a function of device aspect ratio points towards a fixed voltage generation mechanism with current output inversely scaling with the device's internal resistance.

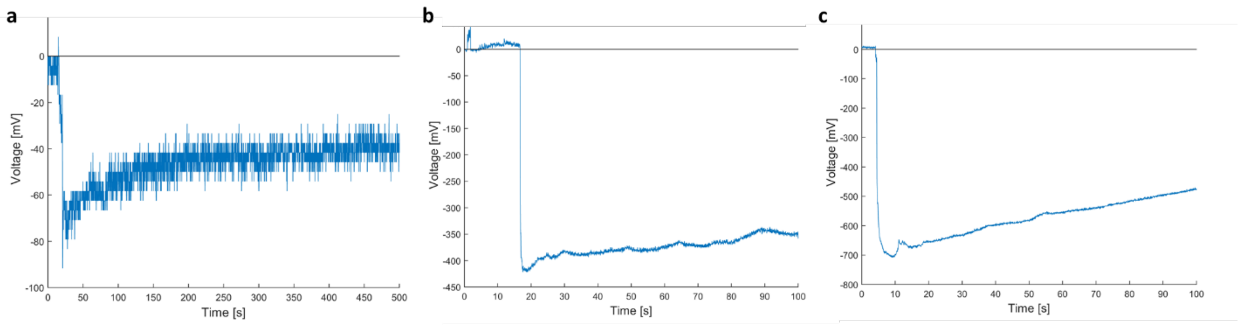

Figure S3-11. Example closed circuit voltage profiles across a 10 kΩ, 200 kΩ, and 5000 kΩ external resistor, measured for an asymmetric device (with Aspect Ratio AR = 2) fabricated using c.a. 25 % [O] oxidized SWNT upon full immersion into CH_3CN. The negative voltage polarity is due to the connectivity of the Janus particle to the oscilloscope. Negative voltage response corresponds to exposed o-SWNT connected with the positive electrode, and vice versa.

3-5. Electron transfer model on Asymmetric Chemical Doping voltage generation

We note that this work aims to provide mechanistic insights of a liquid-solid doping phenomenon (*e.g.,* CH_3CN-SWNT), which involves an electron transfer (ET) process from SWNT to the liquid dopant(s). The resulted species (a radical CH_3CN anion and a hole (h^+), which presumably delocalizes in the SWNT lattice) form **a tightly bound electron-hole pair** due to *Coulombic interactions* (much like that of an exciton), **instead of a** *free* **radical** species (*e.g.,* CH_3CN^-) that *roams* around in the solvent. This actually is how the origin of the term "Asymmetric Chemical Doping" comes about - for we envision this to be analogous to the process in which an intercalated boron atom *p*-dopes a silicon lattice (where the free electrons in the Si lattice migrate and localize in the empty p orbitals of the boron atoms). Only, in our case, we use a liquid *p*-dopant as opposed to a solid-state material that intercalates in the material that is being doped. However, as is the case for boron in silicon, which cannot leave its interstitial location bearing the attracted free electron (as a B^-), the CH_3CN^- should not, in principle, leave the SWNT surface without returning the electron. As we will show, the fact that this radical anion never leaves the SWNT surface actually

plays an important role in the overall energy (or current) generation mechanism. Of course, subsequent electron transfers between “doped” SWNTs (or as the reviewer described “wet SWNTs”) and “undoped” SWNTs are certainly warranted. In fact, this type of ET is instrumental for the observation of an electrical potential between the two electrodes. To be specific, the very reason any ET would occur between a “wet” SWNT and “dry” SWNT is due to the electron withdrawing of the *p*-type liquid dopant from the SWNT in the first place, and this “electron withdrawing” process is seen to be **rate-limiting** and can be described by **a generalized Marcus-type ET theory**.

We first summarize the detailed molecular picture towards the full energy generation process (**Figure S3-12**). We will use CH_3CN as the example *p*-dopant, *without loss of generality*. All details of this tis mechanism described below are now included in the revised manuscript. ***(i)*** Upon CH_3CN adsorption onto the SWNT surface, electrons in the SWNT valence band (*p*-type semiconductor) are transferred to the LUMO of *p*-doping CH_3CN, forming a **tightly-bound $CH_3CN^{\delta-}/SWNT^{\delta+}$** pair. ***(ii)*** Subsequent ET between unexposed SWNTs to the “*p*-doped” or **$CH_3CN^{\delta-}$**-bound region of SWNTs(as a result of the imbalanced Fermi Energies of the SWNT electrons) drives current (h^+) flow from the “undoped” to the “doped” end. ***(iii)*** Dissociation of the **$CH_3CN^{\delta-}/SWNT^{\delta+}$** pairs occurs spontaneously upon (1) thermo-excitation and/or photo-excitation by visible light, which means the electrical energy generated eventually comes from ambient environment, making this phenomenon an energy harvesting scheme. This dissociation process replenishes electrons in the SWNTs, maintaining the overall charge balance, and this “electron withdrawing – thermo/photo excited dissociation” cycle occurs in a dynamic equilibrium, continuously drawing energy from the ambient environment to induce the observed electricity.

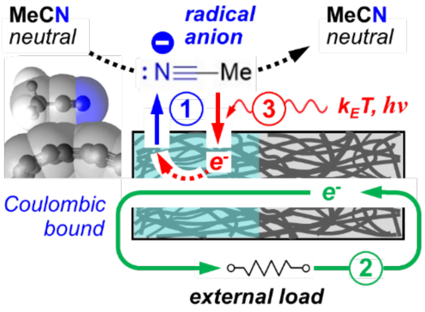

Figure S3-12. Proposed electricity generation mechanism in Asymmetric Chemical Doping, with consideration of the closed circuit current, as well as the fate of the transferred electron.

The interaction of a free electron with a neutral molecule is one of the most fundamental processes in chemistry. Most often, an additional (excess) electron can be accommodated in an empty of half-filled valence orbital of the accepting molecule (VBE, valence bound electron). Alternatively, if the molecule possesses a sufficiently large multipole moment, the excess electron can be trapped in the electric field exerted by the molecule, producing a multipole-bound anion (DBE, dipole-bound electron). Dipole-bound anions appear to be the most abundant, although a number of quadrupole-bound anions have also been reported.

The DBE acetonitrile anion CH_3CN^- can be produced by Rydberg electron transfer[4,5] or by relaxation of charge-transfer-to-solvent (CTTS) excited states of a binary iodide-acetonitrile complex $I^-(CH_3CN)$.[6] The DBE acetonitrile dimer anion $(CH_3CN)_2^-$ has also been synthesized by photoexcitation and CTTS relaxation of the ternary iodide-acetonitrile cluster $I^-(CH_3CN)_2$, and the resulting dimer anion was postulated to have a linear head-to-tail structure $NCCH_3$---$NCCH_3^-$. The lowest unfilled valence π*(C–N) orbitals of acetonitrile are relatively high in energy (ca. 2.8 eV higher than the highest occupied molecular orbital),[7] and the VBE acetonitrile radical-anion CH_3CN^- exists only as a metastable species in the gas phase.[8] Experimental studies of γ- or X-ray irradiated solid[9,10] and liquid[11,12] acetonitrile suggest that, in a polar medium, the CH_3CN

molecules bind an excess electron into valence orbitals. Two distinct species can be produced in solid acetonitrile, depending on the crystal structure: the $(CH_3CN)_2^-$ dimer is formed in α-acetonitrile, whereas the monomeric radical anion $(CH_3CN)^-$ is formed in β-acetonitrile.[13] Since CH_3CN^- readily reacts with a neutral CH_3CN molecule to form $(CH_3CN)_2^-$, it can only be observed in solid β-acetonitrile, whose crystal structure precludes dimerization. In liquid acetonitrile, an excess electron may exist as either a VBE in $(CH_3CN)_2^-$ like in solid acetonitrile or a classical solvated electron in dynamic equilibrium. The latter is a separate entity in a solvent cavity, which is stabilized by the aggregate field of the solvent and can be considered as a condensed-phase analog of the DBE. Photoelectron spectroscopy studies of negatively charged acetonitrile clusters $(CH_3CN)_n^-$ have shown that the DBE and VBE forms coexist for cluster with n = 11-100, but for clusters with n >=13, the VBE form prevails.[14,15] DFT calculations suggest that, for n = 4-6, the VBE form becomes thermodynamically stable.[16] Interestingly, the experimentally observed addition of a hydrogen atom to an acetonitrile molecule inside water cluster anions has been proposed to involve transfer of the excess electron to the acetonitrile molecule and subsequent reaction of CH_3CN^- with a water molecule.[17]

More recently, researchers have started to examine the ultrafast relaxation dynamics of excess electrons injected into liquid acetonitrile.[18,19] Unlike water, when CH_3CN molecules bend, their electron affinity increases, so that a molecular anion can be stabilized in which the excess electron forms a covalent bond between the cyano-carbons of two bent, antiparallel CH_3CN molecules. Thus, when excess electrons are introduced into liquid CH_3CN, two species are formed.[20,21] One of these species has an absorption spectrum in the near-IR that is identical to that of solvated electrons in solvents with similar polarity,[22] and this species has been assigned to be a typical DBE. The assignment of the other species, which absorbs weakly in the visible region of the spectrum,

has been controversial, but current consensus suggests that this entity is a solvent-stabilized VBE $(CH_3CN)_2^-$. Time-resolved photoelectron spectroscopy experiments by Neumark and co-workers found a greater population of the weakly bound species for small $(CH_3CN)_n^-$ clusters and more of the deeply bound species in larger clusters.[23-25]

This brief literature review suggests that the acetonitrile radical anion does form quite readily in liquid form, and CH_3CN could potentially be considered as an electron sink for SWNTs, which certainly plays a key part of our proposed mechanism.

To probe the existence of this “unpaired” electron in the doped SWNT sample, we performed Electron Paramagnetic Resonance Spectroscopy (EPR) at room temperature, according to the following procedure. A Bruker EMX spectrometer is used, with an ER 4199HS cavity and a Gunn diode microwave source producing X-band (8-10 GHz) radiation. Four experiments are performed with 4 mm EPR tubes after sample tuning: A) with liquid CH_3CN, B) with solid HiPCO-SWNT powder, C) with in situ injection of CH_3CN into the SWNT powder, and D) with in situ injection of propanol into the SWNT powder (**Figure S3-13**). The purpose of the first two experiments is easy to explain, checking whether there are pre-existing radical species within the sample. Experiment C) serves as the proof-of-concept, checking whether radical species are indeed formed upon acetonitrile injection. Experiment D) serves as a negative control, in that we had previously observed and reported (in the manuscript) that propanol generates negligible voltage, and should, in fact, generate undetectable levels of radical species.

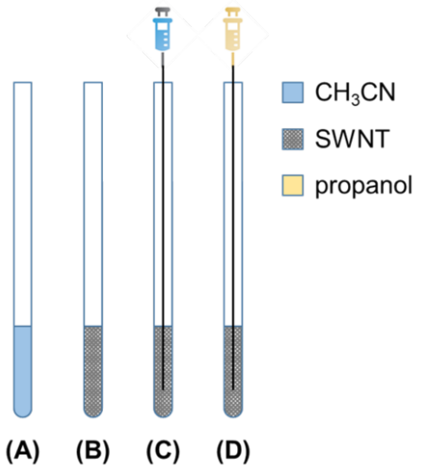

Figure S3-13. Schematic showing which EPR experiments are performed.

We summarize the preliminary results of the EPR analyses here (**Figure S3-14**). We feel these data strongly support our proposed mechanism of the generated CH_3CN^- radical anion Basically, at room temperature, there exists a broad EPR signal for only experiment C), which is shortly (< 30 s) after CH_3CN is injected into the HiPCO-SWNTs. We attribute the broad signal to (i) this experiment being performed at a relatively high temperature (298 K), (ii) SWNT powder being a strong microwave absorber, resulting in insufficient sample tuning before the experiment, and perhaps most importantly (iii) there being a plethora of SWNTs with different chiralities and orientations, and therefore, different kinds of CH_3CN^-/(*n*, *m*)-SWNT pairs, as seen by the EPR producing a broad signal.

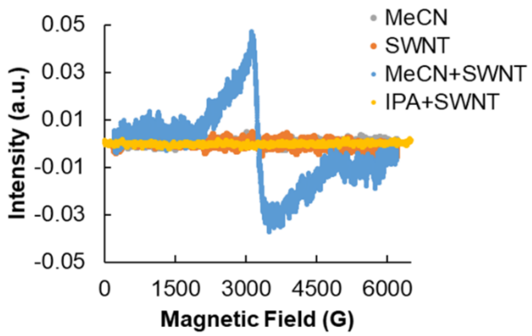

Figure S3-14. EPR spectrum of A) liquid CH_3CN, B) solid HiPCO-SWNT powder, C) in situ injection of CH_3CN into the SWNT powder, and D) in situ injection of propanol into the SWNT powder.

Now that we have shown the existence of an unpaired electron exclusively in a voltage generating MeCN/SWNT system, we would like to assess whether we are generating free radicals or are these dipole-bound anion species tightly adhering to the SWNT surface, presumably due to Coulombic interactions? To answer this question, two experiments are designed.

First, we reasoned that if there are free radicals around the solution, and these free radicals are important for the voltage generating process, then if we use a radical inhibitor to "capture" these radicals, the electricity generation process should be affected. To this end, we performed open-circuit-voltage (V_{OC}) measurements for pristine HiPCO-SWNT flakes using (i) pure CH_3CN (control group) and (ii) 0.5 M Mequinol (MEHQ, a common organic radical inhibitor) dissolved in CH_3CN. The results for doping experiments performed on 24 sets of devices (12 devices for each type of dopant) are summarized in **Figure S3-15**. The black circles are actual data points, whereas the red squares stand for the averages (84.5 ± 8 mV for MeCN, and 86.0 ± 12 mV for MEHQ solution). The fact that excess radical inhibitors seem to not have affected the electricity generation process suggests that there are not any free radicals in solution. Rather, the formed radical anions of acetonitrile should be tightly bound to the SWNT surface.

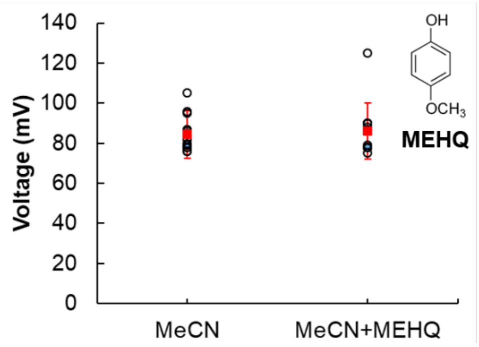

Figure S3-15. Open circuit voltages plotted against (i) acetonitrile (ii) 0.5 M MEHQ dissolved in acetonitrile as dopants for 24 pristine HiPCO-SWNT flake devices tested.

The second sets of experiment we performed are using the radical chain polymerization of ethylene glycol dimethacrylate (EGDMA) as a probe reaction to test whether there are any free radicals in solution (**Figure S3-16**). We reasoned that if there is an appreciable amount of free radicals in solution, one should observe the rapid chain polymerization of EGDMA. The experiments are performed as follows: after filtering out the radical inhibitors in the EGDMA monomer and acetonitrile N_2 purging (to get rid of the residual O_2 for potential radical inhibition), we prepare 3 reaction types: (i) acetonitrile + EDGMA only (negative control), (ii) acetonitrile + AIBN + EDGMA (positive control), and (iii) acetonitrile + SWNT + EDGMA. All three samples are prepared in triplicate to avoid potential experimental error, and we performed the polymerization in two types of general conditions (oil bath and sonication at 40 °C).

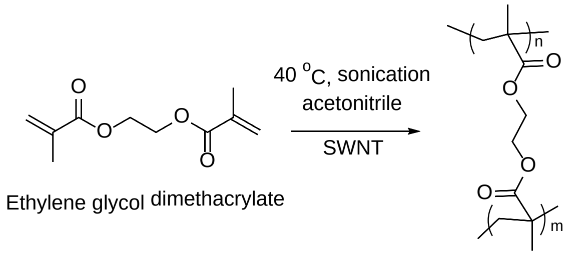

Figure S3-16. Reaction scheme of radical chain polymerization of ethylene glycol dimethacrylate (EGDMA) in SWNT/acetonitrile mixture.

Of the 18 reactions ran (2 types of heating × 3 types of samples × 3 triplicates), we only saw polymerization in the 6 AIBN added samples (positive controls). For the SWNT in acetonitrile reactions, not only did we not observe any weight gain to the SWNTs (due to absorbed/grafted polymer), we did not detect any sign of poly-EGDMA in solution or on SWNT surface (verified

by Raman spectroscopy). This is another strong indication that the SWNT/CH_3CN doping system does not generate any free radical in solution that is potentially useful for chain polymerization.

The dissociation mechanism of the CH_3CN^- radical anion has been deeply investigated experimentallyever since it was first discovered. As early as the 1968 *Nature* report, it has been established that the photo-excitation of the $(CH_3CN)_2^-$ radical anion can be readily reversed via thermal means.[13] Later, researchers established that both the $(CH_3CN)_2^-$ radical anion and the multimer anion (solvated electron in acetonitrile) can undergo photo-excited fragmentation back to its neutral state.[11] It has been reaffirmed that the dipole-bound radical anion species can lose the negative charge and return to the bulk solvent, both through thermal and photo- excited means.

We launched most of the experiments in dark condition as much as possible to avoid unexpected effect of ambient light. The ferrocene oxidation was done in wells covered by aluminum foil, as well as experiments in Figure 3. The reaction systems were only briefly exposed to light when taking photos and doing UV-vis characterization. To test whether the tightly-bound CH_3CN^- /SWNT pair can dissociate with visible light irradiation, we performed the following dark room short-circuit measurement: while the SWNT flake is generating a short-circuit current inside a dark chamber, we pulse laser in the visible/UV range to the flake, and see if any photo-induced current is generated (**Figure S3-17**). Using a tunable filter, we were able to scan through a large range of excitation wavelengths. We programmed the laser such that it shines onto the SWNT sample for 30 s then off for 30 s before it switches to another wavelengthand repeats the process.

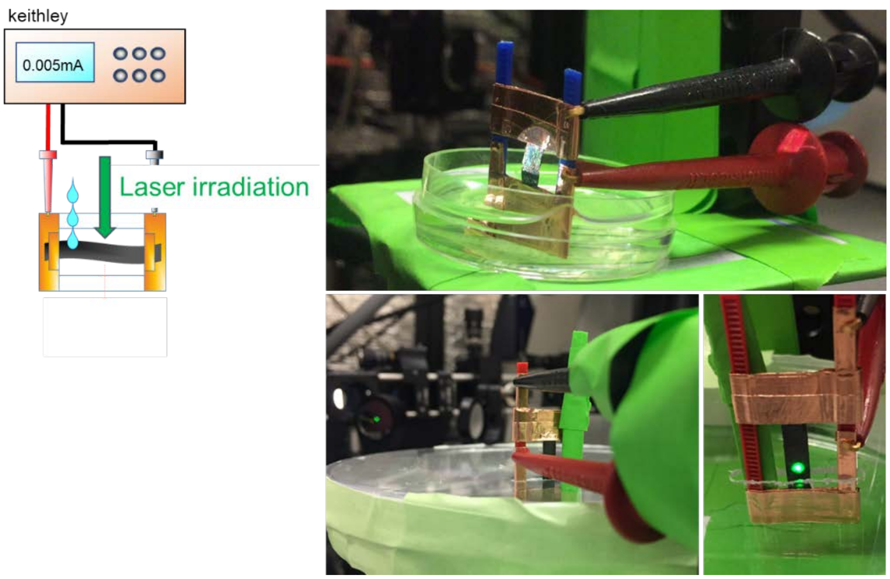

Figure S3-17. (Left) Schematic illustration of the experimental setup. (Right) Pictures during the experiment, in which the current generating SWNT flake is being irradiated with pulsing UV-VIS range tunable wavelength laser.

The results we obtained are very interesting (**Figure S3-18, S3-19**). First of all, we note that the current being generated by the Asymmetric Chemical Doping (ACD) process is around 0.2 μA, and the pulsatile laser irradiation has caused local perturbation to the generated current, leading to the "saw-toothed" pattern (**Figure S3-18**). If we assign the ACD generated current as the baseline current, and subtract that out from the combined measurement, we obtain the pure "photonic" contribution to the current generation process (**Figure S3-19**). Even though detailed analysis needs to be performed for this photo-current to yield quantitative information of our system, it is obvious that photo-excitation can result in perturbations of the current generation process, which we attribute to the increased rate of CH_3CN^-/SWNT pair dissociation.

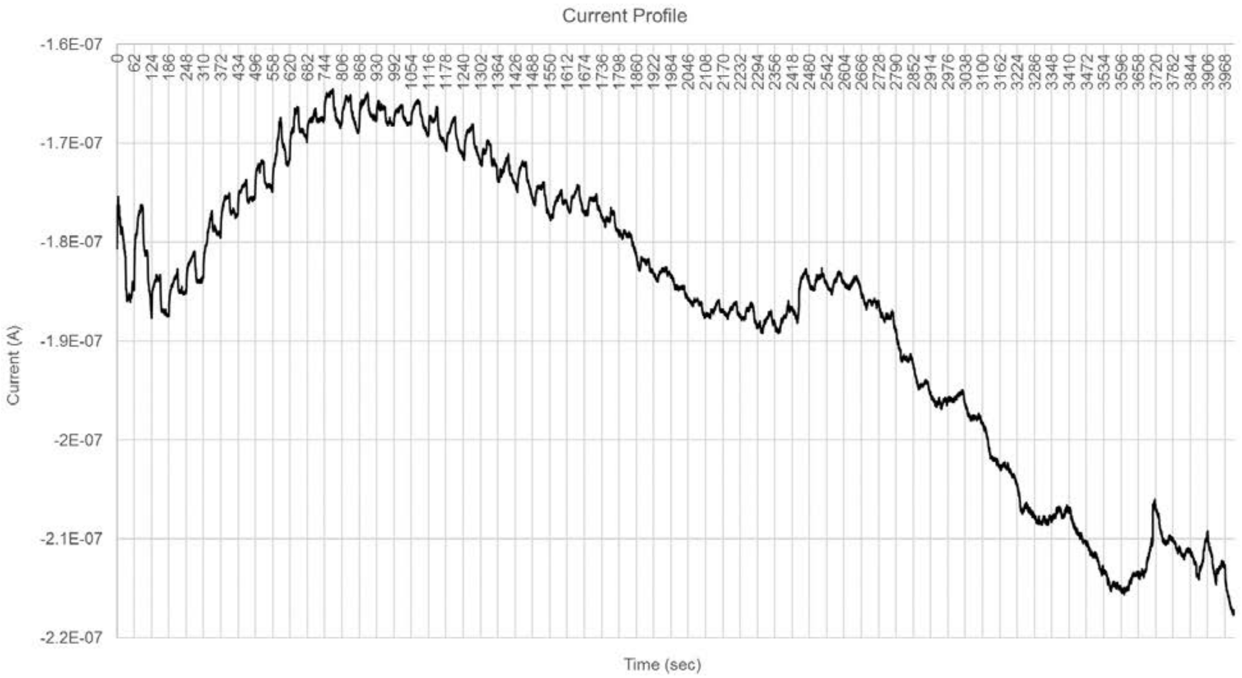

Figure S3-18. Raw short circuit current profile for ACD electricity generation in dark with pulsing laser irradiation.

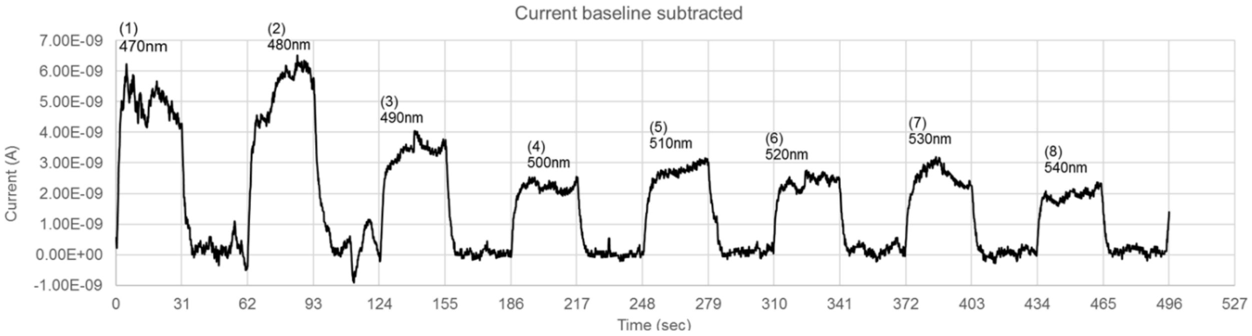

Figure S3-19. Baseline ACD current subtracted short circuit current profile for ACD electricity generation in dark with pulsing laser irradiation (every 30 s, each time of laser pulse and corresponding laser wavelength is labeled on top of the current profile).

4. Validation of equivalency between large and small particles on the experiment of Ferrocene oxidation

In standard particle fabrication, we compress vacuum dried o-SWNTs in a hot press (at 50 °C) with a polymer backing to yield a Janus sheet of polymer protected o-SWNT (**Fig. S4-1a**). Further dicing of the sheet into rectangular shape gives rise to Janus particles of different aspect ratios. The depth of the particles (or the o-SWNT/polymer sheets) are controlled via packing density of o-SWNT prior to the hot-pressing step. We can dice the particles to small pieces commensurate to the width of a human hair (**Fig. S4-1b**). These smaller particles (volume ≈ 360 μm (length) × 200 μm (width) ×100 μm (depth) = 7.2×10^6 μm^3) exhibit the same SWNT and polymer micro structures seen in their larger counterparts (4 mm^3 volume, **Fig. S4-1c)**. When subjected to the same reaction conditions with TBAP as electrolyte and Ferrocene in CH_3CN as solvent, the smaller particles (560) works just as well as one larger particle (same overall o-SWNT volume) in turning over the oxidation reaction (**Fig. S4-1 d, e**). It is worth noting that the smaller particles appear to be have faster kinetics of oxidation of ferrocene to ferrocenium (**Fig. S4-1e**), and this can be attributed to the increased overall surface area for the smaller particles than the larger one. Overall the yield of the reaction is quantitative for both cases.

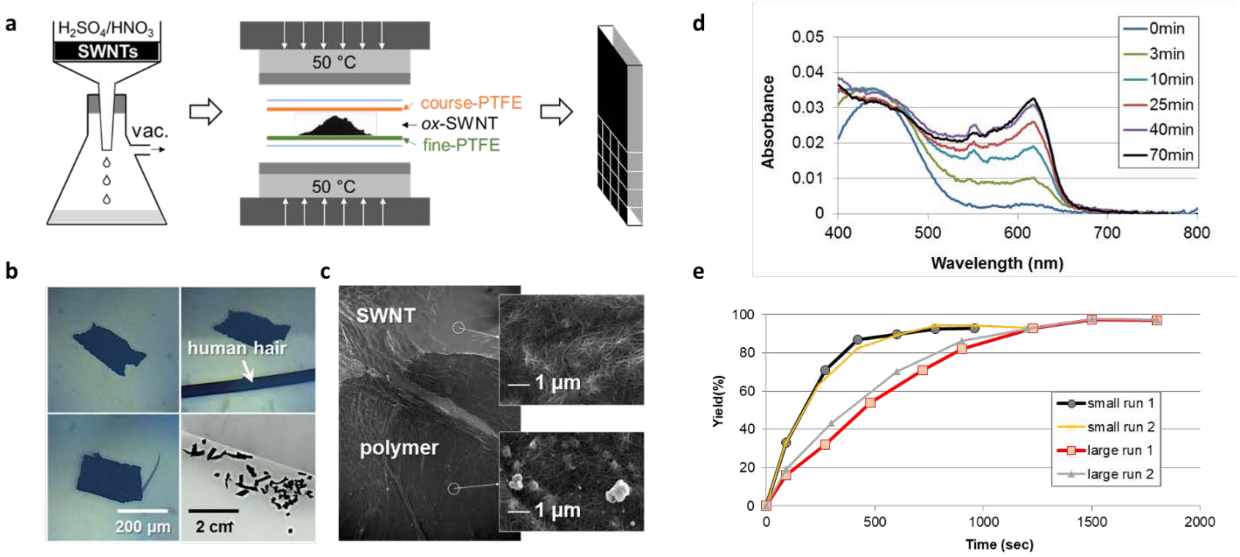

Figure S4-1. (a) Schematic illustration of the procedure of making o-SWNT/PTFE particles. The course-PTFE surface is used to prevent the oxidized SWNTs from sticking to the surface of the hot press (or the insulating layer) that helped the Janus o-SWNT/fine-PTFE sheets to be peeled off. (b) Optical micrography of Janus o-SWNT/PTFE sheets diced into finer Janus particles. Scale bars are 200 µm for top-left, top-right and bottom-left panel, and is 2 cm for the bottom-right panel. (c) Electron micrograph of Janus o-SWNT/polymer particles with zoomed-in scans detailing the micrographs of the exposed o-SWNT and polymer protected o-SWNT section. Scale bars, 1 µm. (d) UV-Vis spectrum of ferrocene reaction mixtures presented as a function of time, the peak around 600 nm corresponds to the oxidized ferrocenium product. (e) Calibrated ferrocene oxidation yield as a function of time for both the small (560 7.2 × 106 µm^3 particles) and large (4 mm^3) particle system. The kinetic data for two consecutive runs are shown.

5. Kinetics and rate limiting step analysis

5-1. Assessment of the role of o-SWNT surface catalysis

Detailed kinetic analyses reveal that the initial rate of the electrochemical process is limited by ferrocene adsorption on the anodic (exposed) o-SWNT surface of the Janus particles. We used UV-Vis kinetic data to back out the ferrocene oxidation reaction mechanism (a catalytic process on the o-SWNT surface). We considered a three-step reactant (ferrocene) adsorption – surface

reaction – product (ferrocenium) desorption model and use experimental means to decide which step is rate limiting and use subsequent fittings to elucidate kinetic parameters.

Without the presence of a catalyst, the ferrocene ↔ ferrocenium redox pair is an equilibrium reaction. We first asked the question whether a catalytic model even necessary? In other words, can the measured kinetic data be described by a simple 1st order forward and backward reaction?

Suppose A is ferrocene, B is ferrocenium, the measurement we have here is [B] as a function of t:

$$A \rightleftharpoons B \text{ with forward and backward rate constant } k_1 \text{ and } k_{-1} \text{ and } [A_0]=[A]+[B] \tag{0.4}$$

$$\frac{d[A]}{dt} = -k_1[A]+k_{-1}[B] = -k_1[A]+k_{-1}([A_0]-[A]) = k_{-1}[A_0]-(k_1+k_{-1})[A] \tag{0.5}$$

The solution for this reaction system is (with the initial condition $[A](t=0)=[A_0]$):

$$\begin{aligned} [A](t) &= \frac{k_{-1}[A_0]}{k_1+k_{-1}} + [A_0]\left(1-\frac{k_{-1}}{k_1+k_{-1}}\right)\exp\left(-(k_1+k_{-1})t\right) \\ [B](t) &= [A_0]-[A] = [A_0]\left(1-\frac{k_{-1}}{k_1+k_{-1}}\right)\left(1-\exp\left(-(k_1+k_{-1})t\right)\right) \end{aligned} \tag{0.6}$$

Now define $\beta \equiv k_1 + k_{-1}$, and the equilibrium constant $K_{eq} \equiv \frac{k_1}{k_{-1}} = \frac{[A]_{eq}}{[B]_{eq}}$, one can do some algebra and rewrite the time dependent ferrocenium (product) concentration profile using only a single kinetic parameter β:

$$[B](t) = [A_0]\left(1-\frac{\beta}{(1+K_{eq})^2}\right)\left(1-\exp(-\beta t)\right) \tag{0.7}$$

In this expression, there is only a single fitting parameter β, with all the other ones easily measured or calculated. We plotted the kinetic data (blue circles) and used MATLAB to perform least square customized equation fitting (**Fig. S5-1**). The three exponential fits are: $[B](t) = p_1 \exp(p_2 t) + p_3$, (3-parameter fit), $[B](t) = p_1 \exp(p_2 t)$ (2-parameter fit), and a one-parameter fit that follows the derived equation exactly. As we can see, while both the three- and two-parameter exponentials fit the kinetics data fairly well, the one-parameter fit is very poor and cannot describe the measured kinetics. This analysis in a good indicator that the mechanism of Janus particle assisted ferrocene-oxidation is a Janus particle assisted process and cannot be described without considering the catalytic surface of the o-SWNTs that make up the Janus particle.

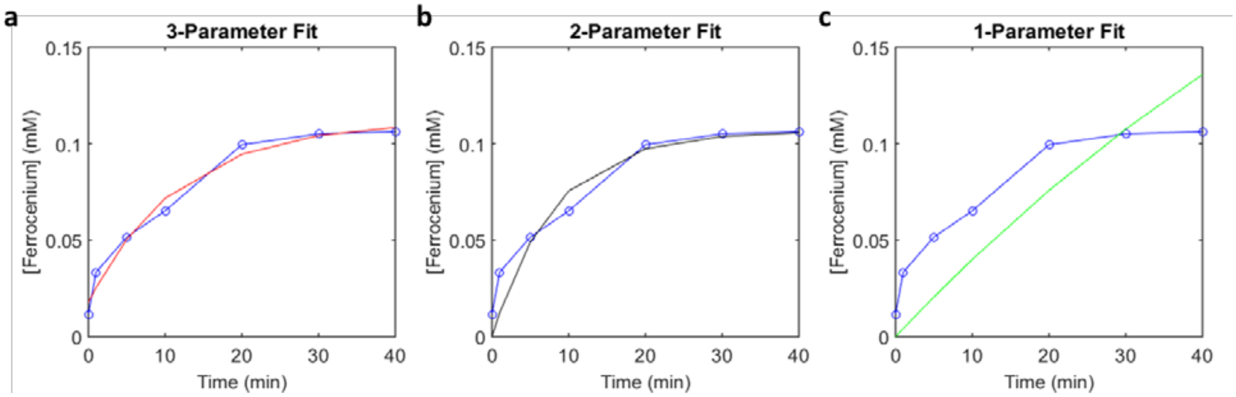

Figure S5-1 Experimental (UV-Vis) kinetic data (blue circles) on ferrocerium (product) concentration plotted as a function of time, along with exponential rate law fits using three (a), two (b) and one (c) parameters.

5-2. Derivation of rate laws for three distinct limiting cases (adsorption, reaction, or desorption)

In order to determine the role the o-SWNT surface plays in the catalytic electro-oxidation of ferrocene, we proposed detailed adsorption – reaction – desorption mechanisms and derive the corresponding rate laws for each of these mechanisms.

The catalytic reaction process (on the anodic surface) can be treated as three consecutive steps:

$$A + S \rightleftharpoons AS \text{ with forward and backward adsorption rate constant } \mathrm{k}_A \text{ and } \mathrm{k}_{-A} \tag{0.8}$$

$$AS \rightleftharpoons BS + e^- \text{ with forward and backward surface reaction rate constant } \mathrm{k}_S \text{ and } \mathrm{k}_{-S} \tag{0.9}$$

$$BS \rightleftharpoons B + S \text{ with forward and backward desorption rate constant } \mathrm{k}_D \text{ and } \mathrm{k}_{-D} \tag{0.10}$$

If we define equilibrium constants K_A, K_S, and K_D the same way we did K_{eq}, the rate of these three equilibrium rates can be express as:

$$r_A = k_A\left([A][S] - \frac{[AS]}{K_A}\right), \text{ where } K_A \equiv \frac{k_A}{k_{-A}} \tag{0.11}$$

$$r_S = k_S\left([AS] - \frac{[BS][e^-]}{K_S}\right), \text{ where } K_S \equiv \frac{k_S}{k_{-S}} \tag{0.12}$$

$$r_D = k_D\left([BS] - K_D[B][S]\right), \text{ where } K_D \equiv \frac{k_{-D}}{k_D} \tag{0.13}$$

At steady state, there is no accumulation of reaction species on the anodic surface, that is $r_A = r_S = r_D$. Here we consider three scenarios that takes each of these steps as rate limiting (while the other two are still in equilibrium). The motivation is that we know from prior analysis that these steps cannot all be in equilibrium with each other (which would have recovered a single parameter exponential concentration profile.

If adsorption if rate limiting, k_A is small compared to k_S and k_D, therefore $\frac{r_S}{k_S} \approx \frac{r_D}{k_D} \approx 0$. This when combined with previous derivation, gives:

$$[AS] = \frac{[BS][e^-]}{K_S} \text{ and } [BS] = K_D[B][S] \quad (0.14)$$

Which in turn yields a rate expression for the limiting adsorption step:

$$r_A = k_A\left([A][S] - \frac{K_D[B][S][e^-]}{K_A K_S}\right) \quad (0.15)$$

At this point, we performed a site balance on the o-SWNT surface:

$$[\theta] = [S] + [AS] + [BS] = [S] + \frac{K_D[B][S][e^-]}{K_S} + K_D[B][S] \quad (0.16)$$

Where [θ] marks the total number density of adsorption sites on the o-SWNT surface. From this we can solve the number density of empty sites [S] as:

$$S = \frac{[\theta]}{1 + \frac{K_D[B][e^-]}{K_S} + K_D[B]} \quad (0.17)$$

And the corresponding adsorption rate law as:

$$r_A = k_A\left(\frac{[\theta]}{1 + \frac{K_D[B][e^-]}{K_S} + K_D[B]}\right)\left([A] - \frac{K_D[B]}{K_A K_S}\right) \quad (0.18)$$

The initial rate for the adsorption limited mechanism, at which $[B] = [e^-] = 0$, and $[A] = [A]_0$, can be reduced to:

$$r_A^i = k_A[\theta][A]_0 \tag{0.19}$$

This means that if we plot the initial rate as a function of the reactant initial concentration, an adsorption limited mechanism would yield a linear dependence.

If, on the other hand, the surface reaction is rate limiting, similar analysis yields:

$$r_S = k_S\left(\frac{[\theta]}{1+K_A[A]+K_D[B]}\right)\left(K_A[A]-\frac{K_D[B][e^-]}{K_S}\right) \tag{0.20}$$

The initial rate for the reaction limited mechanism, at which $[B]=[e^-]=0$, and $[A]=[A]_0$, can therefore be reduced to:

$$r_S^i = \frac{k_S K_A[\theta][A]_0}{1+K_A[A]_0} \tag{0.21}$$

In other words if we plot the initial rate as a function of the reactant initial concentration, an adsorption limited mechanism would yield a plateaued increase dependence.

Lastly, for the desorption limiting scenario, the analysis yields:

$$r_D = k_D\left(\frac{[\theta]}{1+K_A[A]+\frac{K_S K_A[A]}{[e^-]}}\right)\left(\frac{K_S K_A[A]}{[e^-]}-K_D[B]\right) \tag{0.22}$$

The initial rate for the desorption limited mechanism, at which $[B]=[e^-]=0$, and $[A]=[A]_0$, can therefore be reduced to:

$$r_D^i = k_D[\theta] \tag{0.23}$$

In other words if we plot the initial rate as a function of the reactant initial concentration, an desorption limited mechanism would yield no dependence of the initial rate on the initial reactant concentration.

5-3. Experimental validation of rate law of ferrocene electro-oxidation

In the previous section we developed an analytical model of how the initial rate of ferrocene electro-oxidation would depend on the initial ferrocene concentration. In this section, we used UV-Vis spectrometer to perform the initial rate kinetic studies (**Fig. S5-2a**). Different concentrations of ferrocene were prepared, so were the Janus o-SWNT/polymer particles. The kinetics measurements were performed as soon as the baking of the particles are done. Samples were kept under innert (N_2) environment for maximum reproducibility. Before each measurement, we recorrect the UV-Vis baseline using pure CH_3CN, scan the whole spectrometer range to locate the forrocene and ferrocenium peaks. We the initial rate measurements, we take 622 nm (ferrocenium wavelength of the absorption peak) absorbance repeatedly at 2 Hz. We always start spectrum collection before inserting the particle into the quartz cuvette. For measurements over two minutes, the cuvette is covered with a plastic film to prevent evaporation of solvent evaporation.

As seen from the initial rate data as a function of the initial reactant concentration (**Fig. S5-2b**), the initial rate follows a linear trajectory within the concentration window of interest. This indicates that the o-SWNT Janus particle assisted ferrocene oxidation is rate limited by the adsorption of ferrocene onto the anodic o-SWNT surface. This experiments further indicates that the reaction rate can be accelerated by increasing surface are of the particles (and hence its

accessibility to ferrocene). This finding supports our explanation to the observation (Section 4) that smaller Janus particles with equivalent volume (but increased overall surface area) perform the oxidation of ferrocene towards the same yield but at faster rates.

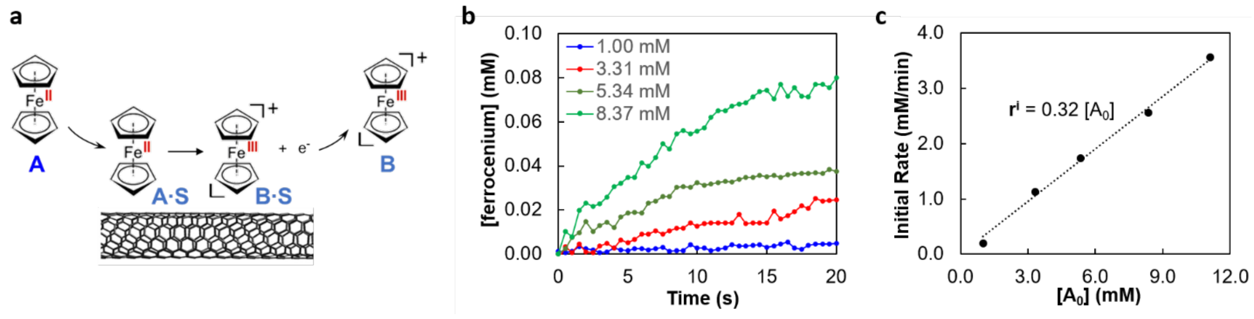

Figure S5-2 (a) Experimental (UV-Vis) kinetic data on ferrocerium (product) concentration plotted as a function of time for different initial ferrocene concentrations. (b) Initial ferrocenium production rate as a function of initial ferrocene concentration, along with a linear fit that suggests an adsorption limited surface catalytic mechanism.

6. Ferrocene-polymer oxidization details

6-1. Polymerization

Ferrocene-polymer was polymerized from Ferroenyl methylmethacrylate (FMMA) using free radical polymerization. FMMA was purchased from Sigma-Aldrich and used as purchased. 400 mg of FMMA and 6 ml of toluene were mixed in the flask filled with N_2 for 30 min using bubbler, then 238 mg of AIBN (Sigma-Aldrich) was added. During 4-hour polymerization, the solution was kept stirring at 85 °C in N_2 atmosphere. After the polymerization, poly-FMMA (PFMMA) was purified by precipitation method 2 times using diethyl ether as a poor solvent. M_w = 80526, M_n = 45778, M_w/M_n = 1.76 were obtained by GPC characterization (**Fig. S6-1a**).

6-2. Polymer coating on SWNTs network

Half of the o-SWNT network was coated by PVA to prevent acetonitrile access, using dip coating method with PVA/water solution. PFMMA was dissolved to THF, and then Janus o-SWNT/PVA network was coated with PFMMA solution using dip coating method and dried at room temperature. The dip coating – drying process was repeated 3 times (**Fig. S6-1b**).

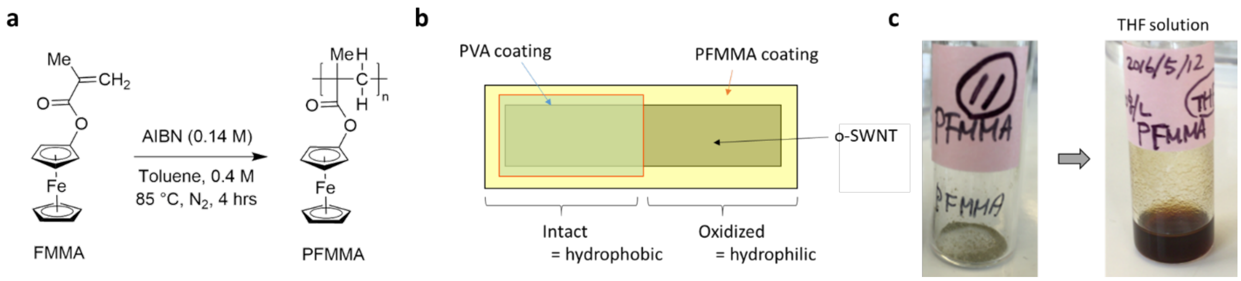

Figure S6-1 (a) Reaction scheme of the radical polymerization of ferroenyl methylmethacrylate. (b) Schematic illustration of the Janus o-SWNT network fabricated to test whether the oxidation of ferrocene (anchored to PFMMA backbone to resolve spatially specific information) occurs on the anodic side (exposed o-SWNT) or the cathodic side (PVA protected o-SWNT). According to our hypothesis, only the PFMMA tethered on the anodic surface will be oxidized (hence the polymer become hydrophilic) while the cathodic side PFMMA will remain in its reduced (hydrophobic) state. (c) Optical image of PFMMA dissolved in THF (before used to coat the o-SWNT/PVA Janus particle).

6-3. Characterization of PFMMA oxidation state

To further explore the detailed mechanism of the ferrocene–to–o-SWNT electron transfer, and how it couples with the driving ACD process, we investigate on which side of the o-SWNT/PVA Janus particle does the ferrocene oxidation occur. This was probed by grafting a thin layer of poly-ferrocenylmethyl-methacrylate (PFMMA) over the entire particle surface and subjecting the PFMMA-grafted o-SWNT/PVA into a CH_3CN/electrolyte solution. Since the only ferrocene

molecules available for electrochemical oxidation are covalently linked to the PFMMA backbone, they are spatially fixed to the particle surface.

We took advantage of the spatial resolution of X-ray Photoelectron Spectroscopy (XPS) to analyze the PFMMA coated Janus o-SWNT/PVA particles after immersion of the particle into CH_3CN for 30 minutes (**Fig. S6-2a**). As always, extensive baking (25 minutes at 200 °C) was performed prior to each experiment to avoid any acid contamination (e.g., H_2SO_4, HNO_3) to the o-SWNT assisted oxidation of ferrocene moieties fixed spatially in PFMMA.

We first performed a control study, in which PFMMA (Fe^{2+}) was chemically oxidized to the oxidized (Fe^{3+}) version of PFMMA ("PFMMA-ox") using HNO_3. Both PFMMA (blue) and PFMMA-ox (red) were examined under XPS (**Fig. S6-2b**). We temporarily assign the 706 eV binding energy peak to the Fe(II)2$p_{3/2}$ orbital in PFMMA and the peak around 718 eV to the Fe(II)2$p_{1/2}$ orbital (**Fig. S6-2b**, blue). The XPS scan for the PFMMA-ox sample, on the other hand, returns a much broadened spectrum with less pronounced features (**Fig. S6-2b**, red). When compared to the reduced PFMMA sample, however, the lack of the previously identified Fe(II)2$p_{3/2}$ orbital peak centered around 706 eV is conspicuous.

With this in mind, we took again a survey scan of the post reaction PFMMA coated Janus o-SWNT/PVA particle with XPS and present the spectrum collected on the exposed anodic o-SWNT side (red) as well as that of the PVA protected side (blue) in **Figure S6-2c**. While both scans appear to be less clean with respect to the control samples, we do observe a 705.5 eV peak for the PVA protected (cathodic) side PFMMA, suggesting perhaps the presence of Fe(II) species in this region. Notably the peak is right-shifted a little when compared to the control sample, potentially due to the conductive carbon layer (o-SWNT) underneath. This 705.5 eV peak, however, is notably

absent from the PMFFA spectrum the exposed (anodic) o-SWNT side, suggesting the lack of Fe(II) species. Instead, the broad peak with higher binding energy than the assigned Fe(II)2p$_{3/2}$ orbital is reminiscent of the Fe(III) feature seen in the PFMMA-ox control sample (**Fig. S6-2c**, red). Admittedly, the XPS analyses did not provide conclusive evidence that the oxidation of PFMMA to PFMMA-ox occurred exclusively on the exposed anodic o-SWNT side of the Janus particle, it however did provide support for the idea that the Janus carbon particle resulted in a spatial asymmetry for the oxidation state of the Fe in the PFMMA layer coated outside of the particle.

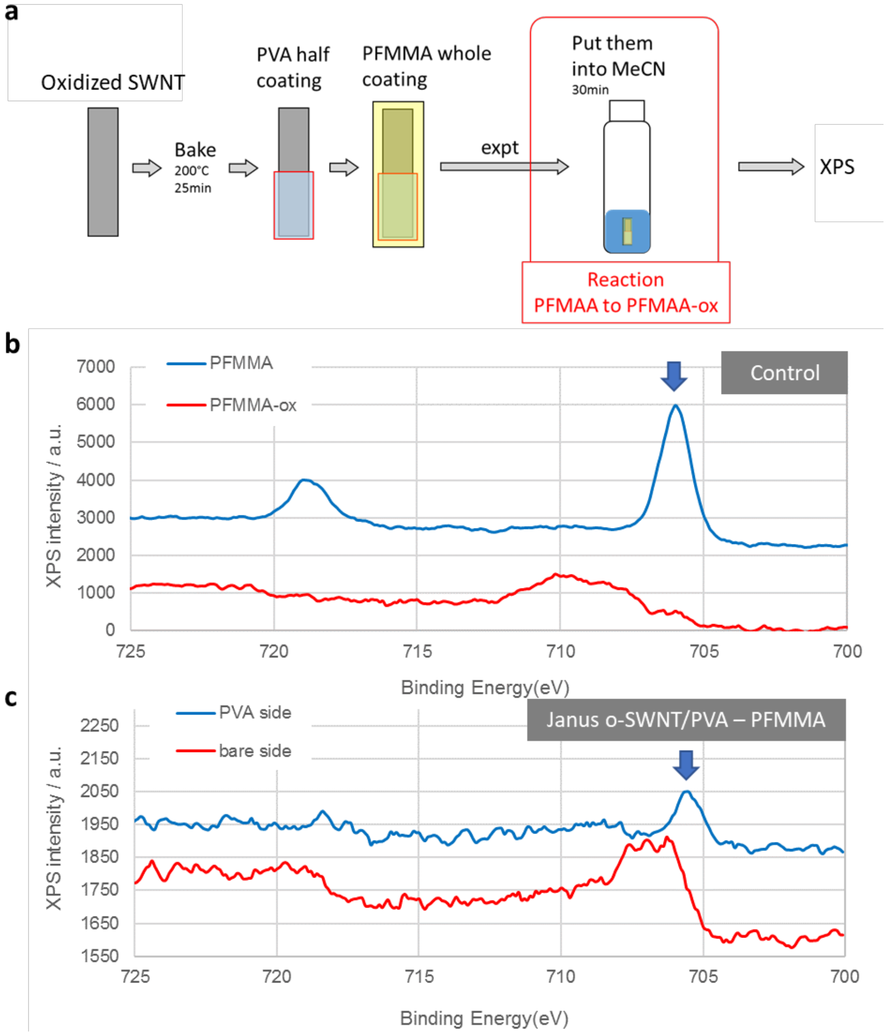

Figure S6-2 (a) Schematic illustration of the post-reaction XPS experimental procedure. (b) XPS survey scan of the control samples (pure polymer, not on Janus particles). PFMMA is blue and PFMMA-ox is red. (c) XPS survey scan of the PFMMA layer on the Janus o-SWNT/PVA particle after immersed (reacted) in CH_3CN for 30 minutes. The PVA side is blue, and the exposed o-SWNT side is red.

In the next set of experiments, we used UV-Vis spectrometry to identify the oxidation state for PFMMA and correlated the PFMMA oxidation state as a function of its location on the Janus o-SWNT/PVA particle. Because UV-Vis measures bulk chemical information without high spatial resolution, we physically cut the PFMMA coated Janus o-SWNT/PVA particle in half (the PVA cathodic half and the exposed anodic half) after its reaction in CH_3CN, extract the PFMMA polymer from the surface of the particle using THF for both sides separately, and analyze their respective oxidation states under UV-Vis (**Fig. S6-3**). As a negative control, we also performed the same particle separation (into two halves), THF extraction, and UV-Vis measurement for PFMMA coated Janus particles that did not go through the CH_3CN immersion (hence ACD mediated electricity generation and subsequent electro-oxidation reaction of ferrocene).

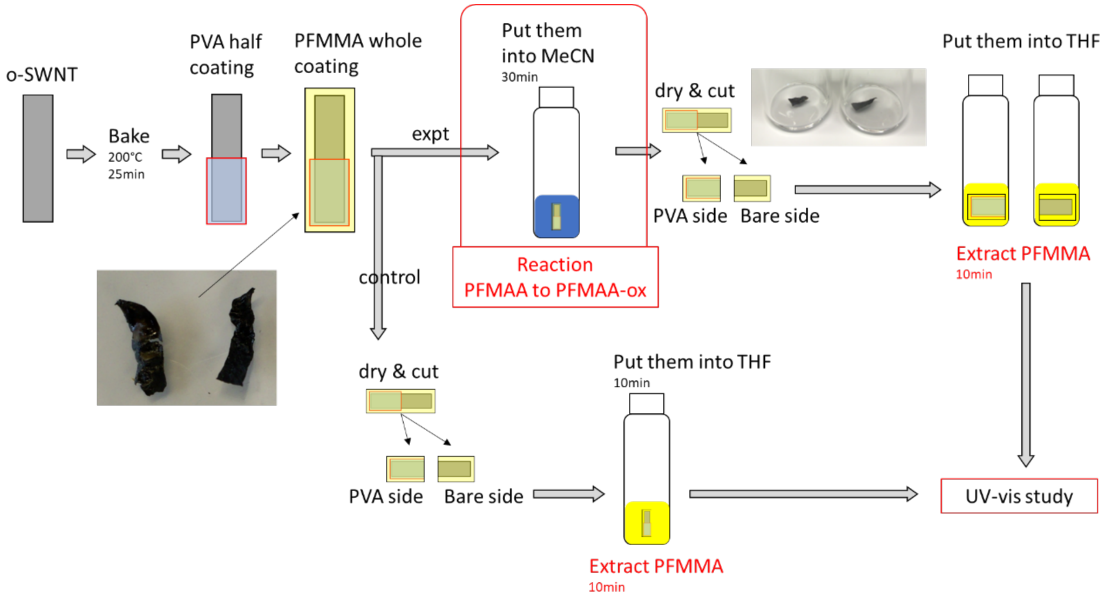

Figure S6-3 Schematic illustration of the post-reaction UV-Vis analyses experimental procedure. Insets are optical images of the Janus o-SWNT particles used in the study.

We first collected the UV-Vis spectra for the as prepared PFMMA (Fe^{2+}) and the chemically oxidized PFMMA-ox (Fe^{3+}) (**Figure S6-4a**). The absorption peak centered around 447 nm wavelength for the reduced PFMMA (Fe^{2+}) is recognized as its spectrometric feature (**Figure S6-4a**, red), which is notably absent from the oxidized PFMMA-ox (Fe^{3+}) sample (**Figure S6-4a**, blue).

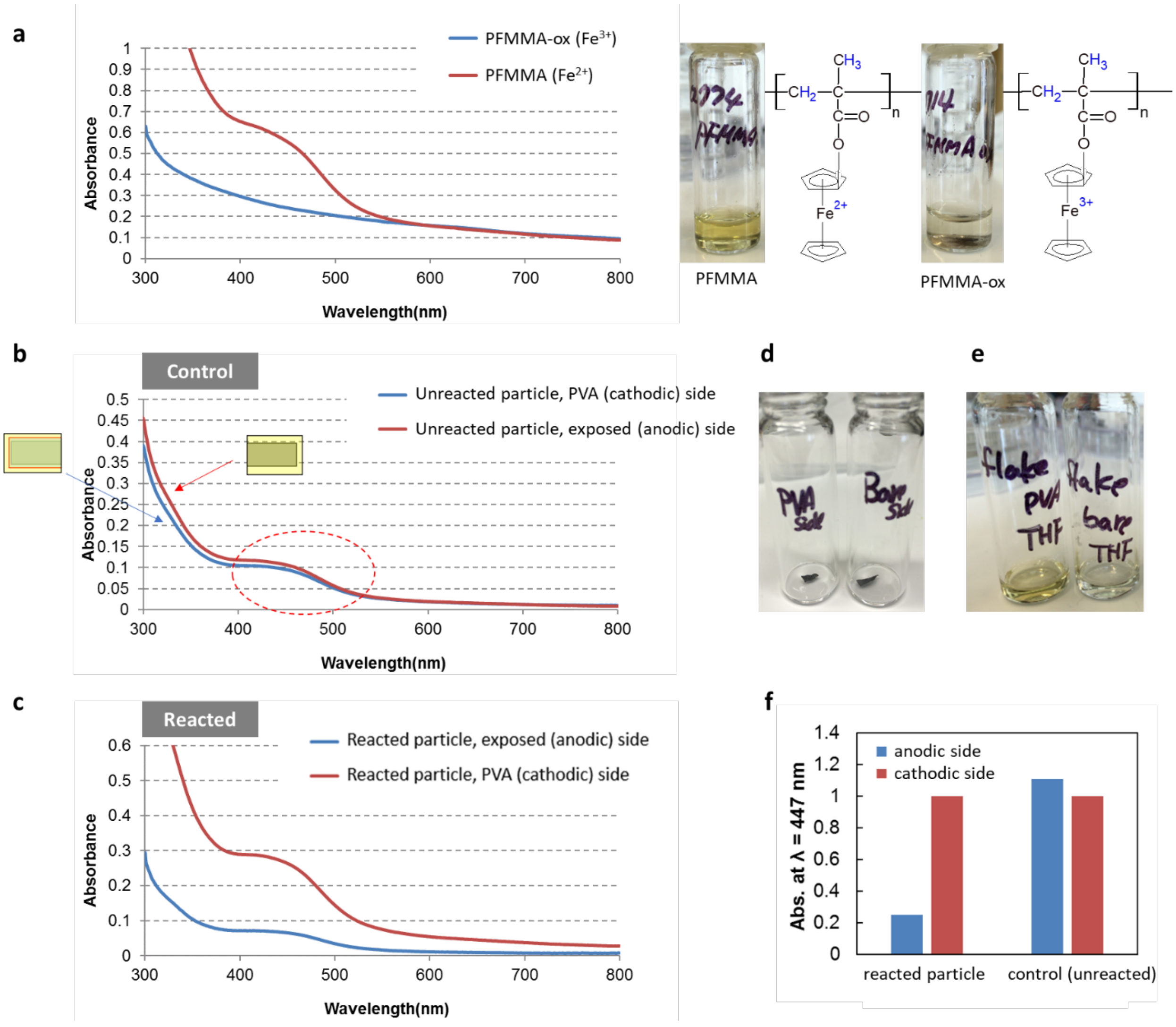

Figure S6-4 (a) UV-Vis spectra for as prepared PFMMA Fe(II) (red) and chemical oxidized PFMMA-ox Fe(III) (blue). Insets are the optical images and chemical structures of PFMMA (left) and PFMMA-ox (right) dissolved in THF. (b) UV-Vis spectra for the control (unreacted) sample, where the PFMMA coated Janus o-SWNT/PVA particle is divided (without reaction in CH_3CN) into a PVA (cathodic) side (blue) and an exposed (anodic) side (red), and each dissolved into a separate THF solution for UV-Vis analyses. (c) UV-Vis spectra for the reacted sample, where the PFMMA coated Janus o-SWNT/PVA particle is divided (upon immersion in CH_3CN) into a PVA (cathodic) side (red) and an exposed (anodic) side (blue), and each dissolved into a separate THF solution for UV-Vis analyses. (d) Optical image of the PFMMA coated Janus o-SWNT/PVA particle divided into a cathodic side (left) and an anodic side (right). (e) THF extracted PFMMA from the cathodic (left) and the anodic (right) side of the PFMMA coated Janus o-SWNT/PVA particle after CH_3CN immersion for 30 minutes (reaction). The color difference between the solutions is discernable by eye. (f) Comparison of the spectroscopic absorbance (at 447 nm wavelength) for both the anodic (blue) and cathodic (red) side of both the reacted PFMMA coated Janus o-SWNT/PVA particle and the unreacted control.

For the PFMMA extracted from the negative control particle, both the PVA protected and the exposed side exhibit the reduced form of PFMMA (**Figure S6-4b**), which is not surprising, because without CH_3CN, no electrical potential will be generated across the Janus o-SWNT/PVA particle, and hence there is no overpotential that drives the thermodynamically unfavorable ferrocene oxidation process. This can also support the electricity mediated oxidation mechanism, for without CH_3CN, a chemically mediated oxidation that directly oxidize the ferrocene moieties in PFMMA should still be possible (and since in our control, the PFMMA coated particle are put in THF to extract the polymer, the lack of solvent to facilitate a direct chemical oxidation should also not be the problem. Also note, THF is known to not generate ACD voltage with o-SWNT, highlighting once more the important role an appropriate molecular dopant such as CH_3CN plays in this electro-oxidation reaction.

After the control group, we characterized particles in the experimental group that was immersed into CH_3CN for 30 minutes (**Figure S6-4c**). Upon reaction, we dry the CH_3CN off, divide the Janus particle into the PVA protected half and exposed (bare) half (**Fig. S6-4c**), and extract the

PFMMA coated on each half with THF (**Figure S6-4d**). It is evident from the color of the THF solution that the PFMMA coated on the PVA protected (cathodic) half is in its reduced form (yellow solution), whereas the exposed o-SWNT (anodic) half has lost that yellow color. This can also be seen in the UV-Vis spectra with a corresponding reduction in the 447 nm absorption peak previously assigned to the reduced PFMMA (Fe^{2+}) species (**Figure S6-4f**), a strong indication of oxidation of PFMMA only occurs on the anodic side of the Janus o-SWNT/PVA particle.

Both the XPS and UV-Vis post-reaction analysis reveals that only the ferrocene molecules bound to the bare o-SWNT side were oxidized into ferrocenium, leaving those on the PVA protected side mostly in their original reduced state. This reaffirms the molecular picture that ferrocene exclusively oxidizes on the electron deficient, unprotected o-SWNT surface (i.e., the anode), driven by the ACD process that lowered the E_F on that side. An interesting corollary of this mechanism states that the electrochemical redox reaction consumes the ACD generated electron flow, and necessarily reduces the solvent-powered closed circuit current, which we explore in the next section.

7. Keythley measurement with changing ferrocene concentration

Short circuit current was measured using Keythley Current Meter with changing ferrocene concentration. Basic setup we used is shown in **Figure S7-1a**. Keytheley Current Meter was connected with Janus particle in series, which was soaked in the prepared MeCN solutions with different concentration of ferrocene from 0 to 20 mM. The resulted current trend in **Figure S7-1c**, **d** clearly shows that the short circuit current through the Janus particle decreases as concentration of ferrocene increases. This indicates that the short circuit current resulted from the

MeCN doping is a measure of free electron available to perform the chemical reaction as shown in **Figure S7-1b**, and its magnitude directly competes with the reaction rate.

We believe the reduced current in the external circuit should be completely due to the increased rate of redox reaction. Any continuous DC current flowing through an electrolyte must be Faradic, and must correspond to redox reaction on electrodes. The only possible non-Faradic DC current is the capacitive current in the electrical double layer, which is minimum in long time. Therefore the reduced current in Fig. 3a is almost exclusively due to increased consumption from redox reaction.

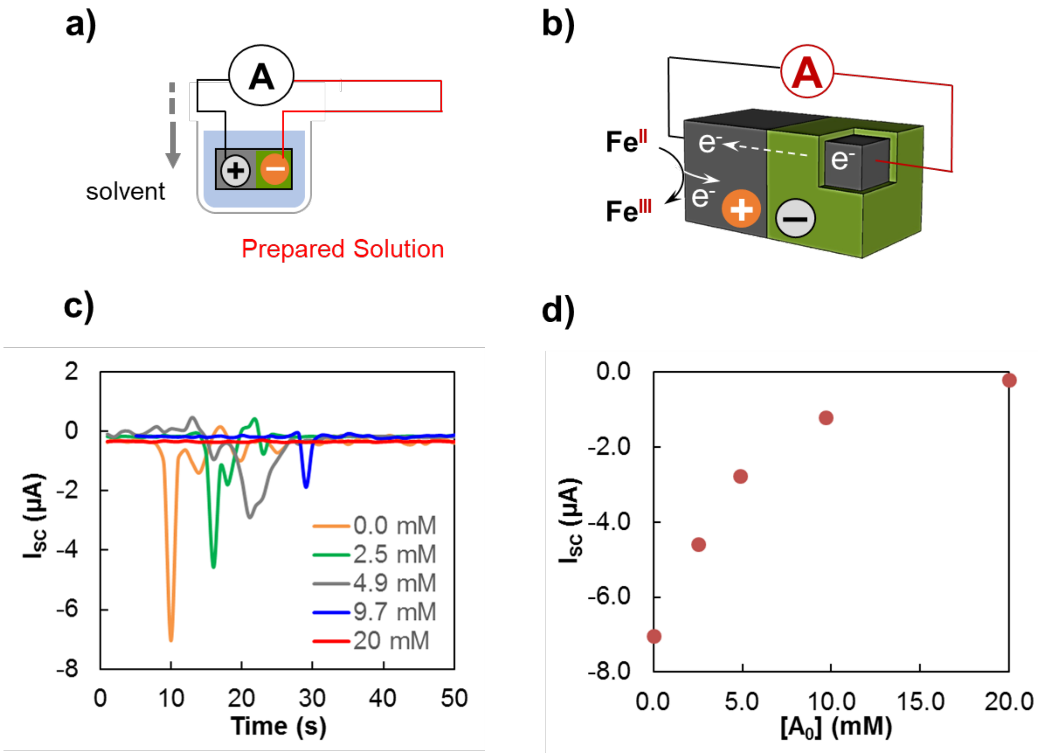

Figure S7-1 (a) Schematic illustrations of current measurement set up dipping in the prepared solutions. **(b)** Schematic image of electron movement in the Janus particle during the reaction. **(c)** Measured current against time, **(d)** Peak intensity shows a clear trend against concentration of ferrocene

8. CV(redox potential) of ferrocene derivatives

Redox potentials of ferrocene derivatives, discussed in Figure 3 in the main text, were measured using Cyclic Voltammetry (CV) technique. The MeCN solution was prepared with adding 50 mM of t-bap as an electrolyte, and then 1 mM of each ferrocene derivative, listed in **Table S8.1.**, was added to prepare the solution for CV measurement. The measurement was scanned by 50 mV/s, using glassy carbon electrode and Ag/AgCl electrode for working and reference electrode, respectively. **Figure S8.1(a)** shows the result of CV on Ferrocene (reference), and oxidation potential was determined as 380 mV from its oxidation peak on CV curve. Since Janus particle prepared in this study typically has 500 mV voltage in MeCN, this 380 mV of ferrocene oxidation potential implies it can successfully oxidize ferrocene in MeCN. Oxidation potentials of 9 different Ferrocene derivatives were obtained by same method and are listed in **Table S8-1.**

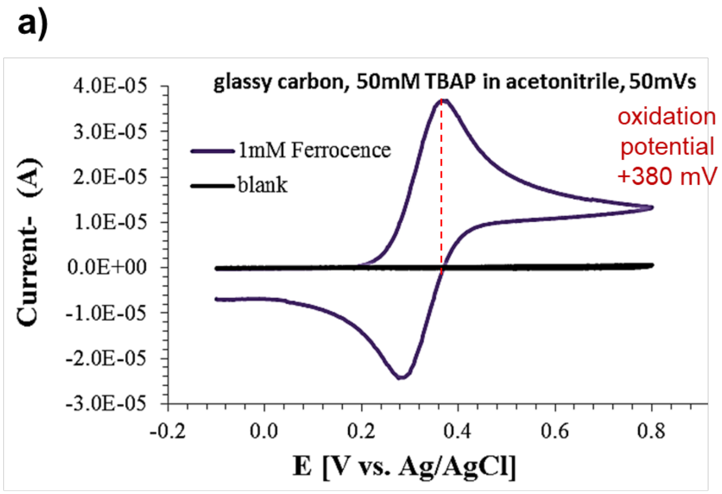

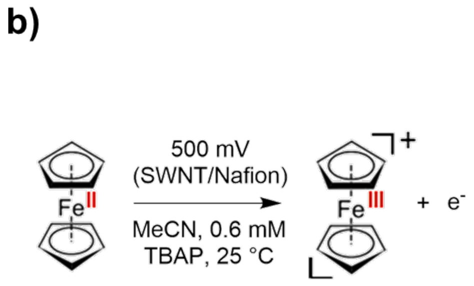

Figure S8.1. (a) CV curve of ferrocene and **(b)** reaction scheme of ferrocene oxidation.

Table S8-1. Oxidation potential of ferrocene derivatives

No	Name of derivatives	Oxidation potential (mV)
ref	ferrocene	380

1	Ferrocene aceticacid	78
2	Ferrocene methanol	114
3	Methylferrocene methanol	195
4	Ferrocene acetonitrile	230
5	6-bromohexyl ferrocene	294.3
6	Ferrocene boronicacid	312
7	Ferrocenyl benzimidazole	383.9
8	Bromooxohexyl ferrocene	466.9
9	Ferrocene dicarboxaldehyde	692.9

9. XPS and SEM of Copper, Cobalt, Ag reduction

9-1. Polymer coating on SWNTs network

Half of the o-SWNT network was coated by Nafion to prevent acetonitrile access, using dip coating method with Nafion solution (as purchased from Sigma Aldrich) at room temperature. The dip coating – drying process was repeated 3 times (Same method with the procedure in S6-2).

9-2. Reaction

Half-coated o-SWNT network was soaked in 3 kinds of metal salts (shown in **Figure S9-1**) in acetonitrile with TBAP(50mM) to launch reactions for 1 hour. During the reactions, metal salts were reduced, as shown in **Figure 3 c)** in the main text, proposed to be adsorbed on the Nafion-coated side of SWNT.

9-3. XPS analysis

In order to identify where actually reduced metal salts are, metal atomic concentrations on both sides of o-SWNT and Nafion-coated SWNT were checked using XPS (ULVAC-PHI, INC. PHI VersaProbe II) with a monochromated Al Kα source. The reduced metal salts content was

calculated as an atomic percentage from the integration of Cu 2p peak, Co 2p peak, and Ag 3d peak, respectively in the high-resolution scans. As shown in **Figure S9-1**, all of three metal salts shows higher concentration on the Nafion-side, which reduction of metal salts occur as explained in the main text. These data strongly support our hypothesis where reduction and oxidation occur on the half-coated SWNT.

a)

Metal Salt	Concentration (mM)	Conversion (%)	Yield (%)
CuCl2	0.6	89.1	39.7
CoCl2	0.6	96.4	24.3
AgCl	0.6	n.a.	43.2

b)

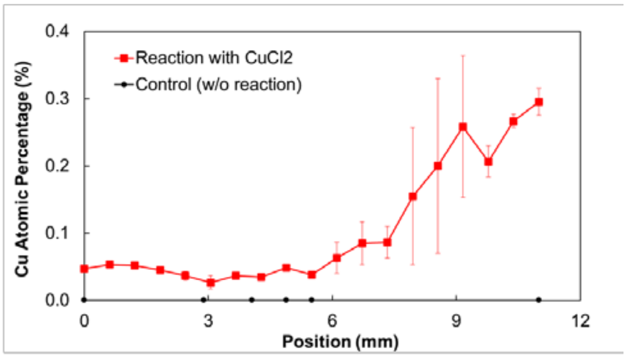

c) d)

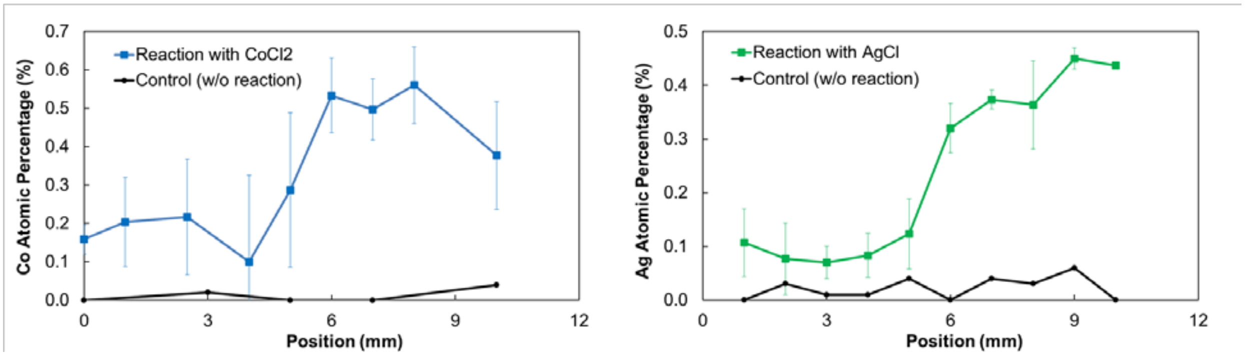

Figure S9-1 (a) Table of metal salts, their concentration used, and conversion, yield obtained in this study. (b) (c) (d) the line profiles of half-coated SWNT surface measured by XPS with CuCl2, CoCl2, AgCl reactions, respectively. Right sides of graphs are the Nafion-coated side and the left side is o-SWNT side.

9-4. SEM analysis

The half-coated SWNT was imaged using a Zeiss Merlin field emission scanning electron microscopy (SEM) and elementally analyzed using an Energy Dispersive X-ray Spectroscopy (EDS). As shown in **Figure S9-2**, there appeared to be a brighter fiber structure on the upper side

due to the Nafion-coating, while there is darker fiber structure on the lower side with bare o-SWNT network. On the bare o-SWNT side, several brighter particles are observed as shown in **Figure S9-2c**. These particles could be Nafion fractions accidentally coated on the bare side during dip-coating process or might be adsorbed during the reaction. These Nafion particles in the o-SWNT side explain the higher existence of reduced metal atoms even within the region of o-SWNT side in the XPS analysis as shown in **Figure S9-1**, comparing to the control o-SWNT. Further EDS analysis in the **Figure S9-2** proves amount of Nafion on the SWNT is important for the reduction of metal salts. There observed darker portion in the left-center of **Figure S9-4.2 a)**, this region should have more Nafion which includes large amount of Fluorine and Sulfur as shown in the elemental maps in **Figure S9-2c, d**. **Figure S9-2b** indicates that this region reduced more copper due to large amount of Nafion, compared to the rest of portion. Thus, these SEM and EDS analyses on the half-coated SWNT elucidate our hypothesis where reduction and oxidation occur furthermore.

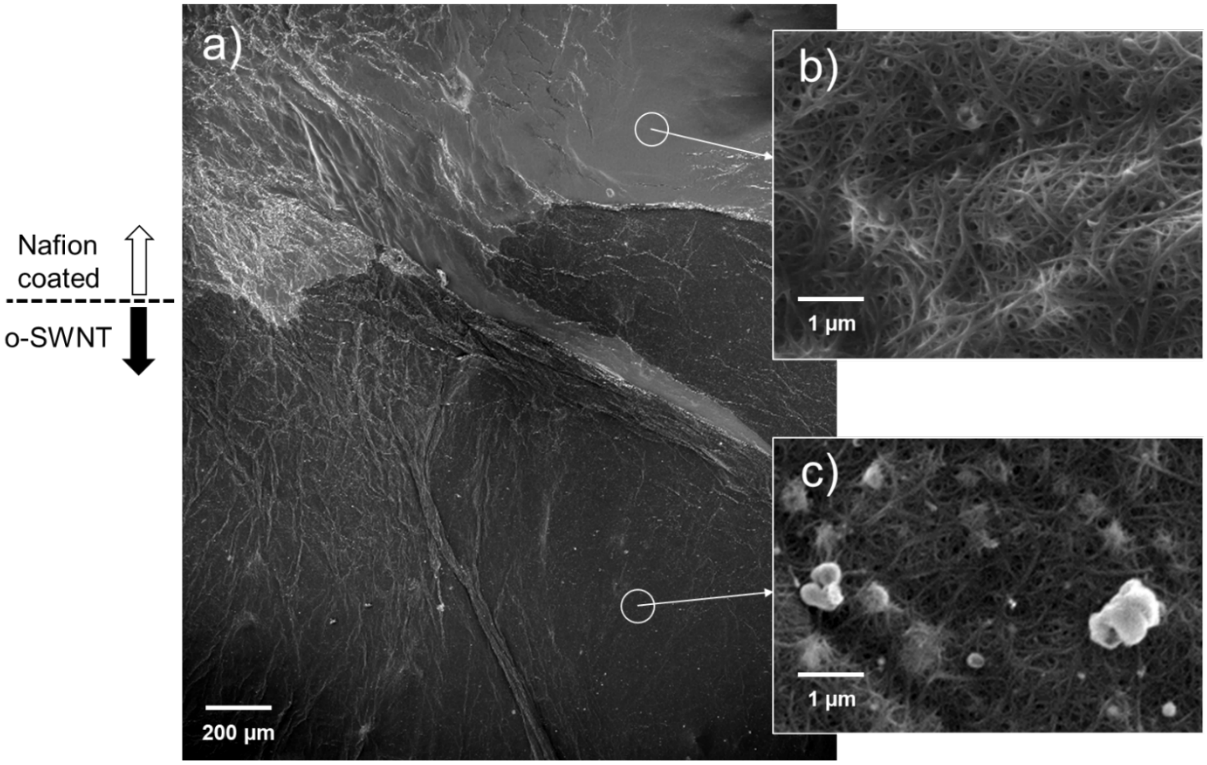

Figure S9-2 a) SEM image of the half-coated SWNT, upper portion is the Nafion-coated SWNT and lower portion is o-SWNTt, rerspectively. Magnified images of b) the Nafion-coated SWNT and c) o-SWNT, respectively.

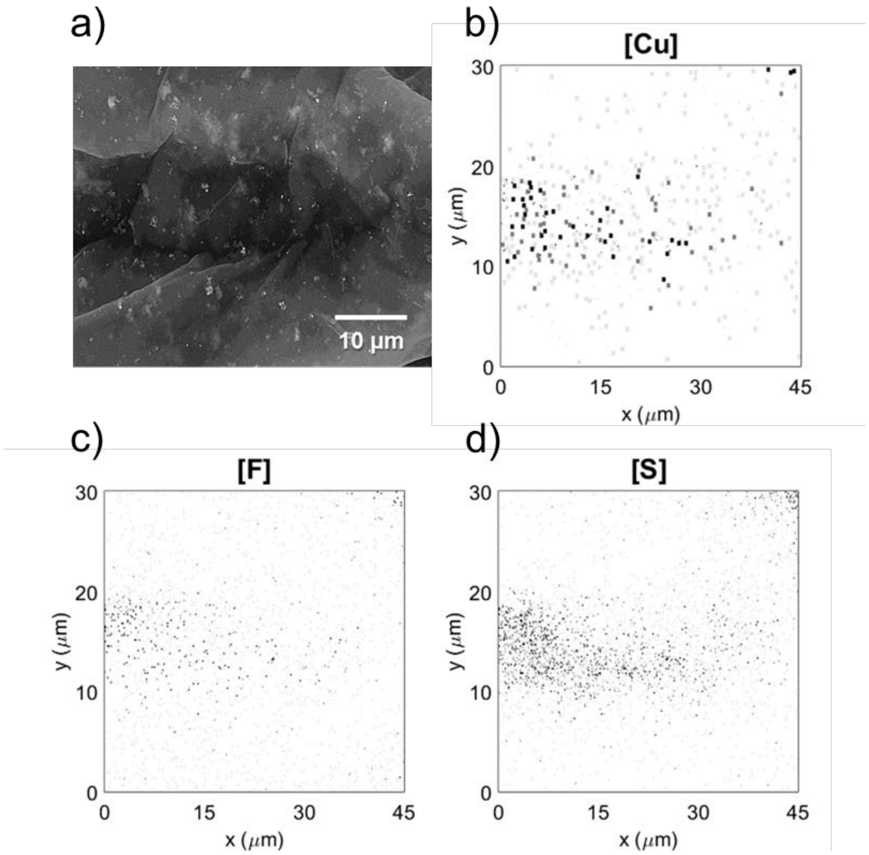

Figure S9-3 a) SEM image of the Nafion-coated SWNT with Nafion concentration gradient on its structure. Darker part in the center has more Nafion with high concentration of Fluorine and Sulfur. b), c), d) are elemental concentration map of Copper, Fluorine, Sulfur, respectively. High concentration of Fluorine and Sulfur indicates there is more Nafion in the center and Copper is observed in the center as well, which means reduction of Copper occurs more in the center part with high concentration of Nafion coating.

10. Procedure of resistance measurement of polymer-coated SWNT

The half-polymer-coated SWNT for the resistance measurement was prepared as followings. Firstly, half portion of the cut SWNTs network was dipped into polymer solution and dry. This procedure is repeated several times to make sure enough thick to prevent MeCN penetration. Then, the edge of polymer coating side is cut diagonally in the thickness direction by razor and cross-sectional naked SWNT is placed on the copper electrode to make a good contact between them.

The other bare side, without any coating, of SWNT is also placed on the copper electrode, bridging across two copper electrodes then were fixed with another copper.

Resistance across SWNT was measured through copper electrodes by multi-tester with two-probe method.

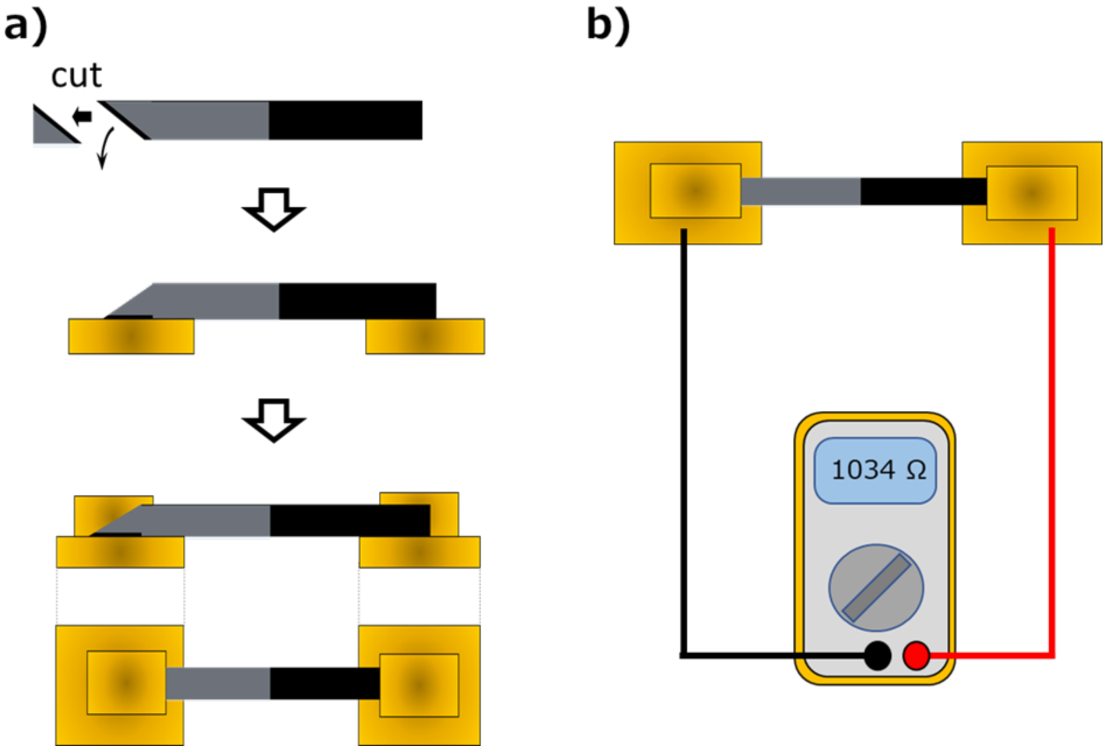

Figure S10-1 a) The schematic procedure of half-polymer-coated SWNT device for resistance measurement. All the schematic except bottom one is cross-sectional view of SWNT and electrodes. Top indicates SWNT is cut diagonally in the thickness direction on order to make sure good contact with the electrode. b) The schematic of resistance measurement using multi-tester with two-probe method.

11. Hypothesis on mechanism of long-lasting voltage creation in the reaction solution.

Although ADC voltage creation lasts only for a few seconds when electrons are consumed as an electrical current doing some work, ie., flow the external circuit, observed electrochemical reaction, in the main text, lasts for 30 minutes or more. In order to understand this long-lasting phenomenon, we propose a hypothesis to give an adequate explanation for this long-lasting reaction phenomenon here.

At a first process, as we propose in the previous works, voltage creation occurs due to the asymmetric MeCN doping on SWNT which creates Fermi level gap in SWNT, leading the potential bias across the SWNT(**Figure S11-1a**) with negative voltage on the bare SWNT side.

Then secondly, this voltage makes chemicals reacted electrochemically as shown in **Figure S11-1b**. Since this reaction puts electrons on the bare SWNT side and takes electrons from PTFE SWNT side which makes voltage decrease, these chemical reactions should have occurred quickly and voltage disappeared in short time without extra mechanism to sustain voltage. However, in our experiment, most of the reactions went for a long time more than 30 minutes, so here we propose one additional mechanism concomitantly from our ADC scheme in electrochemical reactions written in the main text.

As shown in **Figure S11-1b**, once Ferrocene oxidation occurs on the bare side of SWNT, counter reaction, hydrogen generation, occurs on the other side as well. When this counter reaction goes further, there creates a $COOH/COO^-$ gradient across the SWNT as shown **Figure S11-1b**. Liu et al., have showed a long-lasting current generation using protein and gold electrodes. In their work, they proposed that the water gradient from surface to bottom, created

due to its asymmetric structure, brings the proton gradient due to the ionization of carboxylic acid and concomitant diffusion of proton, which drives a long-lasting electric current on their system. Similar phenomenon should happen in our system, because there should be a $COOH/COO^-$ gradient after a while reaction goes as shown in **Figure S11-1b**. This $COOH/COO^-$ gradient promote a proton diffusion across the SWNT through hopping of proton on COOH. During the proton diffusion, bare SWNT (left) side of **Figure S11-1c**) loses protons leaving negative charge on COO^- behind, and on the other hand, PTFE (right) side obtains extra protons with positive charge. This creates the same direction of potential bias with the bias created by original ACD doping, eventually keeping the voltage high for a longer time compared to the voltage observation without these reactions.

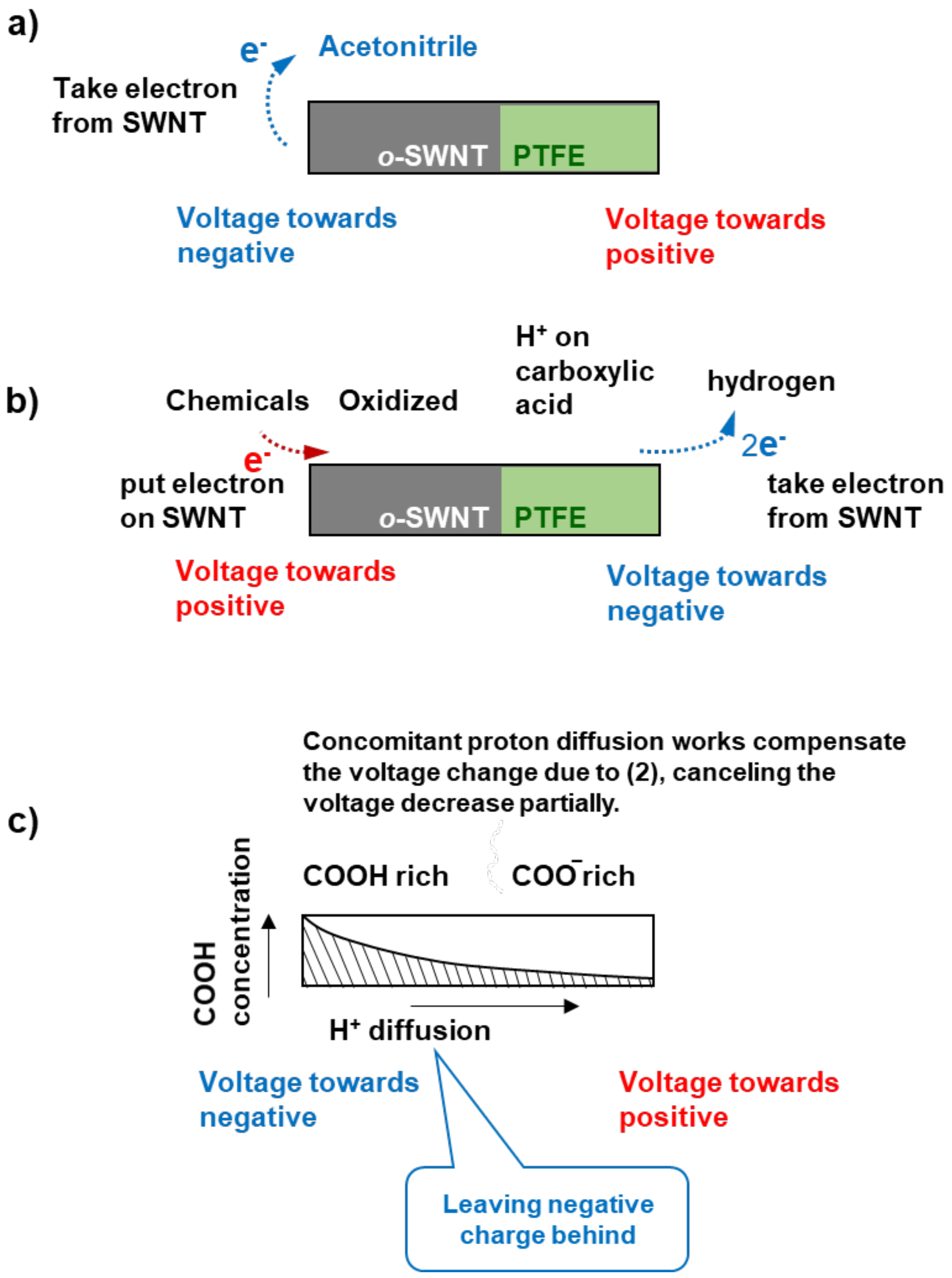

Figure S11-1. Schematics of a) Voltage creation mechanism and its voltage direction based on ADC theory, b) Electro-chemical reaction and its direction of voltage consumption, c）Concomitant proton diffusion with electro-chemical reaction of b), canceling voltage decrease by leaving negative charge on COO^-.

(1) Tvrdy, K.; Jain, R. M.; Han, R.; Hilmer, A. J.; McNicholas, T. P.; Strano, M. S. *ACS Nano* **2013**, *7*, 1779.

(2) Marulanda, J. M.; Srivastava, A. *physica status solidi (b)* **2008**, *245*, 2558.

(3) Kunai, Y.; Liu, A. T.; Cottrill, A. L.; Koman, V. B.; Liu, P.; Kozawa, D.; Gong, X.; Strano, M. S. *Journal of the American Chemical Society* **2017**, *139*, 15328.

(4) Stockdale, J. A.; Davis, F. J.; Compton, R. N.; Klots, C. E. *The Journal of Chemical Physics* **1974**, *60*, 4279.

(5) Desfrançois, C.; Abdoul-Carime, H.; Khelifa, N.; Schermann, J. P. *Physical Review Letters* **1994**, *73*, 2436.

(6) Dessent, C. E. H.; Bailey, C. G.; Johnson, M. A. *The Journal of Chemical Physics* **1995**, *103*, 2006.

(7) Jones, R. O.; Seifert, G. *The Journal of Chemical Physics* **1992**, *96*, 7564.

(8) Bailey, C. G.; Dessent, C. E. H.; Johnson, M. A.; Jr., K. H. B. *The Journal of Chemical Physics* **1996**, *104*, 6976.

(9) Williams, F.; Sprague, E. D. *Accounts of Chemical Research* **1982**, *15*, 408.

(10) Shkrob, I. A.; Takeda, K.; Williams, F. *The Journal of Physical Chemistry A* **2002**, *106*, 9132.

(11) Shkrob, I. A.; Sauer, M. C. *The Journal of Physical Chemistry A* **2002**, *106*, 9120.

(12) Xia, C.; Peon, J.; Kohler, B. *The Journal of Chemical Physics* **2002**, *117*, 8855.

(13) Bonin, M. A.; Tsuji, K.; Williams, F. *Nature* **1968**, *218*, 946.

(14) Mitsui, M.; Ando, N.; Kokubo, S.; Nakajima, A.; Kaya, K. *Physical Review Letters* **2003**, *91*, 153002.

(15) Mbaiwa, F.; Holtgrewe, N.; Dao, D. B.; Lasinski, J.; Mabbs, R. *The Journal of Physical Chemistry A* **2014**, *118*, 7249.

(16) Takayanagi, T.; Hoshino, T.; Takahashi, K. *Chemical Physics* **2006**, *324*, 679.

(17) Balaj, O. P.; Balteanu, I.; Fox-Beyer, B. S.; Beyer, M. K.; Bondybey, V. E. *Angewandte Chemie International Edition* **2003**, *42*, 5516.

(18) Doan, S. C.; Schwartz, B. J. *The Journal of Physical Chemistry B* **2013**, *117*, 4216.

(19) Doan, S. C.; Schwartz, B. J. *The Journal of Physical Chemistry Letters* **2013**, *4*, 1471.

(20) Bell, I. P.; Rodgers, M. A. J.; Burrows, H. D. *Journal of the Chemical Society, Faraday Transactions 1: Physical Chemistry in Condensed Phases* **1977**, *73*, 315.

(21) Timerghazin, Q. K.; Peslherbe, G. H. *The Journal of Physical Chemistry B* **2008**, *112*, 520.

(22) Fox, M. F.; Hayon, E. *Chemical Physics Letters* **1972**, *14*, 442.

(23) Ehrler, O. T.; Griffin, G. B.; Young, R. M.; Neumark, D. M. *The Journal of Physical Chemistry B* **2009**, *113*, 4031.

(24) Ehrler, O. T.; Neumark, D. M. *Accounts of Chemical Research* **2009**, *42*, 769.

(25) Young, R. M.; Griffin, G. B.; Kammrath, A.; Ehrler, O. T.; Neumark, D. M. *Chemical Physics Letters* **2010**, *485*, 59.
